# Genome-Scale Analysis of the *WRI-Like* Family in *Gossypium* and Functional Characterization of *GhWRI1a* Controlling Triacylglycerol Content

**DOI:** 10.3389/fpls.2018.01516

**Published:** 2018-10-16

**Authors:** Xinshan Zang, Wenfeng Pei, Man Wu, Yanhui Geng, Nuohan Wang, Guoyuan Liu, Jianjiang Ma, Dan Li, Yupeng Cui, Xingli Li, Jinfa Zhang, Jiwen Yu

**Affiliations:** ^1^State Key Laboratory of Cotton Biology, Cotton Institute of the Chinese Academy of Agricultural Sciences, Key Laboratory of Cotton Genetic Improvement, Ministry of Agriculture, Anyang, China; ^2^Department of Plant and Environmental Sciences, New Mexico State University, Las Cruces, NM, United States

**Keywords:** cotton, *WRI-like*, expression pattern, cottonseed oil, *GhWRI1a*

## Abstract

Cotton (*Gossypium* spp.) is the most important natural fiber crop and the source of cottonseed oil, a basic by-product after ginning. *AtWRI1* and its orthologs in several other crop species have been previously used to increase triacylglycerols in seeds and vegetative tissues. In the present study, we identified 22, 17, 9, and 11 *WRI-like* genes in *G. hirsutum*, *G. barbadense*, *G. arboreum*, and *G. raimondii*, respectively. This gene family was divided into four subgroups, and a more *WRI2-like* subfamily was identified compared with dicotyledonous *Arabidopsis*. An analysis of chromosomal distributions revealed that the 22 *GhWRI* genes were distributed on eight At and eight Dt subgenome chromosomes. Moreover, *GhWRI1a* was highly expressed in ovules 20–35 days after anthesis and was selected for further functional analysis. Ectopic expression of *GhWRI1a* rescued the seed phenotype of a *wri1-7* mutant and increased the oil content of *Arabidopsis* seeds. Our comprehensive genome-wide analysis of the cotton *WRI-like* gene family lays a solid foundation for further studies.

## Introduction

Cotton, especially upland cotton (*Gossypium hirsutum* L.), is the main source of renewable textile fibers and is also well known for its oil-rich seeds. After ginning, fuzzy cottonseed is processed into four major products: hulls (26%), linters (9%), oil (16%), and meal (45%), with 4% lost in processing (Liu et al., 2009). The most valuable cottonseed oil is typically composed of approximately 26% palmitic acid (C16:0), 15% oleic acid (C18:1), and 58% linoleic acid (C18:2) (Liu et al., 2002). Because cotton is the world’s sixth largest source of vegetable oil (Liu et al., 2002, 2009), increasing cottonseed oil content through classical breeding techniques and biotechnological approaches is important.

Triacylglycerols (TAGs), which accumulate in plant seeds and fruits, are major renewable sources of reduced carbon used as food, industrial feedstocks, and fuel (Bates and Browse, 2012). As reviewed previously (Bates and Browse, 2012), plants use two main pathways to produce diacylglycerol (DAG), the immediate precursor molecule to TAG synthesis. *AtWRI1*, an AP2 transcription factor, is involved in the regulation of seed storage metabolism in *Arabidopsis* (Focks and Benning, 1998; Cernac and Benning, 2004). The homozygous *atwri1* mutant has a wrinkled seed phenotype and exhibits an 80% reduction in seed oil content compared with wild type (WT) *Arabidopsis* (Focks and Benning, 1998; Cernac and Benning, 2004). Expression of *AtWRI1* cDNA under the control of the cauliflower mosaic virus 35S-promoter has been found to lead to increased seed oil content and the accumulation of TAGs in developing seedlings (Cernac and Benning, 2004). The involvement of orthologous genes of *AtWRI1* in the regulation of oil content has also been reported in many other plant species (Liu et al., 2010; Shen et al., 2010; Pouvreau et al., 2011; An and Suh, 2015; Grimberg et al., 2015; Yang et al., 2015; Hofvander et al., 2016; An et al., 2017). For example, in rapeseed, overexpression of *WRI1-like* (*BnWRI1*) cDNAs driven by cauliflower mosaic virus 35S-promoter results in 10–40% increased seed oil content and enlarged seed size and mass in 51 transgenic *Arabidopsis* lines (Liu et al., 2010). In maize, overexpression of *ZmWRI1* results in an oil increase without affecting germination, seedling growth, or grain yield in transgenic maize (Shen et al., 2010; Pouvreau et al., 2011). Eighteen putative target genes of ZmWRI1a have been identified by transcriptomic experiments, 12 of which contain the *cis*-element bound by AtWRI1 in their upstream regions. Interestingly, the higher seed oil content is not accompanied by a reduction of starch in ZmWRI1a transgenic lines, and could be utilized in transgenic breeding (Pouvreau et al., 2011). Recently, expression of *CsWRI1A*, *B*, or *C* has rescued the seed phenotype of the *Arabidopsis wri1-3* loss-of-function mutant (An et al., 2017).

In cotton, *WRI-like* genes are found to be participated in the fiber development and oil seed content. Silencing of the expression of *WRINKLED1* by TRV-VIGS (tobacco rattle virus triggers virus-induced gene silencing), corresponding to *GhWRI1b* in our study, has been found to increase fiber length but reduce oil seed content, suggesting the possibility of increasing fiber length by repartitioning carbon flow (Qu et al., 2012). Fiber transcriptome of *G. hirsutum* producing short-fiber and long-fiber is compared with the transcriptome of extra-long fiber producing *G. barbadense*, and find that expression pattern of a *Wrinkeled1* gene shows close association with fiber length. The authors speculate that *Wrinkled1* transcription factor (GenBank accession number: DW505003.1), also corresponding to *GhWRI1b* in the present study, is involved in the development of extra-long staples in cotton (Qaisar et al., 2017). Moreover, compared with WT, overexpression of *GhWRI1* (GenBank accession number: JX270189), corresponding to *GhWRI1b* in our study, has been observed to increase seed lipid content and decrease protein content in transgenic upland cotton (Liu et al., 2018).

Four *WRI1-like* genes, named *AtWRI1-4*, are present in *Arabidopsis* (To et al., 2012). Seed-specific overexpression of *AtWRI3* and *AtWRI4*, but not *AtWRI2*, can suppress the wrinkled phenotype of *wri1-4* and restore normal oil accumulation (To et al., 2012). These results imply that *WRI-like* family genes play important roles in the developmental regulation of fatty acid and TAG production in plants. In this study, we performed a comprehensive genome-wide analysis to further understand the complexity of *WRI-like* family genes in cotton. In addition, a transgenic approach was used to clarify the function of *GhWRI1a* in TAG production.

## Materials and Methods

### Sequence Retrieval, Multiple Sequence Alignment, and Phylogenetic Analysis

Genome sequences of *G. arboreum* (A2, BGI_V1.0) (Li et al., 2014), *G. raimondii* (D5, BGI_V1.0) (Wang et al., 2012), *G. hirsutum* acc. TM-1 (AD1, NBI_V1.1) (Zhang et al., 2015), and *G. barbadense* acc.3-79 (AD2, SGI_V1.0) (Yuan et al., 2015) were downloaded from the CottonGen website^1^. *AtWRI1*, *AtWRI2*, *AtWRI3*, and *AtWRI4* were acquired from TAIR 10^2^. *WRI-like* genes in cacao were acquired from the cacao genome database^3^. *WRI-like* genes in rice were acquired from the rice annotation project database^4^. To identify *WRI-like* genes from *Gossypium*, AtWRI1, AtWRI2, AtWRI3, and AtWRI4 protein sequences were used as queries against the above-mentioned cotton genomes. ClustalX version 2.0 (Larkin et al., 2007) was used to perform multiple sequence alignments of all identified *WRI-like* genes in this study (**Supplementary File 1**). A phylogenetic analysis was carried out using the neighbor-joining algorithm with the pairwise deletion option, Poisson correction model, and uniform rates, with the statistical reliability of the resulting tree evaluated using 1,000 bootstrap replicates (Tamura et al., 2013). The online ExPASy tool^5^ was used to calculate the sequence length, theoretical molecular weight (MW), and isoelectric point (pI) of WRI-like proteins.

### Chromosomal Location, Gene Duplication, and Gene Loss

MapChart^6^ was used to visualize the mapping of *WRI-like* genes (Voorrips, 2002). Gene duplication events were defined as previously described criteria (Dong et al., 2016; Cui et al., 2017). Gene loss evens were analyzed based on the best match and the syntenic blocks in the CottonGen website^7^. DnaSP software of phylogenetic analysis by the maximum likelihood method was used to calculate *K*a and *K*s of the duplicated gene pairs.

### Genetic Structure Analysis and Protein Domain Detection

*GhWRI* gene structures were generated using the Gene Structure Display Server (GSDS)^8^. The SMART database^9^ was used for detection of GhWRI protein domains.

### Expression Pattern Analysis of *GhWRI* Genes Based on RNA-Seq Data

FPKM values of *GhWRI* genes were calculated using previously reported RNA-seq data of 22 cotton tissues (SRA accession code: PRJNA248163) (Zhang et al., 2015).

### Transgenic Plant Generation and Expression Analysis

The complete coding sequence of *GhWRI1a* (**Supplementary File 2**) was amplified with gene specific primers. The resulting PCR product was cloned into a digested pBI121 vector with BamH I and Sac I using ClonExpress^®^ II One Step Cloning Kit (Vazyme, Nanjing, China). We used *Agrobacterium tumefaciens* strain *GV3101* containing this binary construct to transform *Arabidopsis* plants. Transformants were selected on MS medium supplemented with kanamycin (50 mg/L). The progeny of transformants showed an approximately 3:1 segregation of live and dead phenotypes, and homozygous lines in the T3 generation were used for further analysis (Zang et al., 2017). To detect the relative expression level of *GhWRI1a* in the transgenic *Arabidopsis* lines, siliques were collected 15 days after anthesis (DPA), frozen immediately in liquid nitrogen, and stored at -80 °C for RNA isolation. Quantitative real-time PCR (qRT-PCR) was performed to determine the expression pattern of *GhWRI1a*, with the 2^-ΔΔC^_t_ method (Livak and Schmittgen, 2001) used to quantify the expression level of *GhWRI1a* relative to the 18S rRNA endogenous control. Each experiment was independently repeated in triplicate. Primers are listed in **Supplementary Table S1**.

### Generation of CRISPR/Cas9 Transgenic Plant

For *AtWRI1* gene editing, two single-guide RNAs (sgRNAs) were designed to target the first and fifth exons, namely Target1 and Target2 (**Supplementary Figure S1**). The two integrated targets were ligated to BsaI-digested pRGEB32-GhU6.9 as previously reported (Wang et al., 2017). This construct was introduced into *Agrobacterium tumefaciens* strain *GV3101*, which was used to transform *Arabidopsis* Col-0 as described above. The resulting CRISPR/Cas9 transgenic lines were genotyped for mutations using a pair of primers spanning the two target sequences (**Supplementary Table S1**). The homozygous T3 generation was used for further analysis.

### Oil Content Analysis

We determined total oil content using an NMI20-Analyst nuclear magnetic resonance spectrometer (Niumag, Shanghai, China).

## Results

### Genome-Wide Identification and Phylogenetic Analysis of *WRI-Like* Genes in *Gossypium*

Two diploid cottons, *G. arboreum* (AA genome) and *G. raimondii* (DD genome), evolved from a common ancestor (Zhang et al., 2017). The most widely cultivated tetraploid cotton species are *G. hirsutum* (AADD, AD1 genome) and *G. barbadense* (AADD, AD2 genome), both of which originated from inter-genomic hybridization of two A- and D-genome progenitor species (Paterson et al., 2012). To identify all WRI-like proteins in AD1, AD2, AA and DD genomes, *Arabidopsis* WRI1-4 protein sequences (AtWRI1/AT3g54320, AtWRI2/AT2g41710, AtWRI3/AT1g16060, and AtWRI4/AT1g79700) were queried against reference genomes of the above-mentioned four species. All *WRI-like* candidates were further screened based on the conserved AP2 domain using the SMART database. A total of 59 *WRI-like* genes were identified: 11 in *G. raimondii*, 9 in *G. arboreum*, 22 in *G. hirsutum*, and 17 in *G. barbadense* (**Table 1**). *WRI-like* gene names and identifiers, gene pairs, and predicted properties of WRI-like proteins are listed in **Table 1**.

**Table 1:** Characteristics of *WRI-like* genes and predicted properties of WRI-like proteins.

Family name	Gene name	Gene identifier (NAU)	Chromosomal localization	Size (AA)	MW (KD)	pI
*WRI1*	*GhWRI1a*	Gh_A10G1731	A10	434	48.9534	5.54
	*GhWRI1b*	Gh_D10G2551	D10	435	49.0264	5.54
	*GhWRI1c*	Gh_A13G0020	A13	776	90.3036	8.00
	*GhWRI1d*	Gh_D13G0036	D13	1607	182.1011	8.19
	*GbWRI1a*	Gbscaffold22373.8.1	A10	437	49.3428	5.54
	*GbWRI1b.1*	Gbscaffold14438.14.0	scaffold14438_d10	438	49.4149	5.69
	*GbWRI1b.2*	Gbscaffold14438.14.1	scaffold14438_d10	287	32.5254	4.47
	*GbWRI1b.3*	Gbscaffold14438.14.2	scaffold14438_d10	349	39.4756	8.52
	*GbWRI1c.1*	Gbscaffold18152.14.0	A13	230	25.9051	4.79
	*GbWRI1c.2*	Gbscaffold18152.14.1	A13	228	25.6890	5.01
	*GbWRI1d*	Gbscaffold20501.18.0	A13	390	43.4780	5.91
	*GaWRI1a*	Cotton_A_24703	CA_chr9/A10	437	49.383	5.69
	*GrWRI1a*	Cotton_D_gene_10029828	Chr5/D02	435	49.1105	5.40
	*GrWRI1b*	Cotton_D_gene_10024797	Chr13/D13	394	43.8264	5.69
*WRI2*	*GhWRI2a*	Gh_A02G1061	A02	432	47.8707	8.59
	*GhWRI2b*	Gh_D03G0620	D03	427	47.3351	8.69
	*GbWRI2a*	Gbscaffold3103.5.0	A02	422	46.8407	8.67
	*GbWRI2b*	Gbscaffold1219.3.0	A02	422	46.8257	8.84
	*GbWRI2c*	Gbscaffold9581.6.0	A05	124	14.4788	10.22
	*GaWRI2a*	Cotton_A_37619	CA_chr2/A01	422	46.8267	8.67
	*GrWRI2a*	Cotton_D_gene_10038477	Chr4/D08	419	46.4653	8.69
*WRI3/WRI4*	*GhWRI3a*	Gh_A04G1351	A04	391	44.6319	7.52
	*GhWRI3b*	Gh_D04G0842	D04	361	41.1974	7.25
	*GhWRI3c*	Gh_A05G0024	A05	378	42.8800	7.56
	*GhWRI3d*	Gh_A05G0999	A05	351	39.6935	7.14
	*GhWRI3e*	Gh_D05G0071	D05	378	42.8050	7.86
	*GhWRI3f*	Gh_D05G1117	D05	351	39.6075	7.37
	*GbWRI3a*	Gbscaffold25274.2.0	A04	271	31.1240	9.77
	*GbWRI3b.1*	Gbscaffold1205.6.0	D04	213	24.0384	4.47
	*GbWRI3b.2*	Gbscaffold1205.6.1	D04	363	41.3505	7.27
	*GbWRI3c.1*	Gbscaffold2524.5.0	A05	354	40.0689	7.14
	*GbWRI3c.2*	Gbscaffold2524.5.1	A05	351	39.6935	7.14
	*GbWRI3d.1*	Gbscaffold12660.29.0	D05	354	39.9829	7.37
	*GbWRI3d.2*	Gbscaffold12660.29.1	D05	352	39.6986	6.95
	*GbWRI3e*	Gbscaffold22373.8.0	A10	331	37.7665	4.46
	*GbWRI3f*	Gbscaffold18379.5.0	scaffold18379	261	29.3003	5.64
	*GaWRI3a*	Cotton_A_16267	CA_chr12/A04	370	41.8689	8.29
	*GaWRI3b*	Cotton_A_41232	CA_chr12/A04	364	41.4607	7.00
	*GaWRI3c*	Cotton_A_17105	CA_chr10/A05	351	39.7036	7.14
	*GrWRI3a*	Cotton_D_gene_10016054	Chr9/D05	373	42.1882	7.91
	*GrWRI3b*	Cotton_D_gene_10007101	Chr9/D05	351	39.5725	7.66
	*GrWRI3c*	Cotton_D_gene_10021087	scaffold211	368	41.9301	7.00
*WRI-like*	*GhWRI2-likea*	Gh_D04G0466	D04	432	49.5570	9.44
	*GhWRI2-likeb*	Gh_A05G3160	A05	378	42.5790	7.91
	*GhWRI2-likec*	Gh_A07G1973	A07	365	41.4291	9.63
	*GhWRI2-liked*	Gh_D07G2191	D07	374	42.9831	9.56
	*GhWRI2-likee*	Gh_A09G0218	A09	387	44.4053	8.80
	*GhWRI2-likef*	Gh_A09G0219	A09	278	31.8599	9.73
	*GhWRI2-likeg*	Gh_D09G0206	D09	387	44.2530	8.50
	*GhWRI2-likeh*	Gh_D09G0207	D09	180	20.3753	9.98
	*GhWRI2-likei*	Gh_A12G1529	A12	390	44.1106	9.96
	*GhWRI2-likej*	Gh_D12G1652	D12	387	43.4360	9.84
	*GbWRI2-likea*	Gbscaffold17450.12.0	D04	124	14.5028	10.22
	*GbWRI2-likeb*	Gbscaffold19204.1.0	scaffold19204	381	43.2288	9.96
	*GbWRI2-likec*	Gbscaffold1804.8.0	scaffold1804	365	41.3300	9.54
	*GbWRI2-liked*	Gbscaffold259.12.0	scaffold259	387	43.4620	9.77
	*GaWRI2-likea*	Cotton_A_29003	CA_chr12/A04	378	42.5830	7.91
	*GaWRI2-likeb*	Cotton_A_19437	CA_chr9/A07	320	36.3994	9.99
	*GaWRI2-likec*	Cotton_A_28204	CA_chr11/A09	386	44.1049	8.52
	*GaWRI2-liked*	Cotton_A_06134	CA_chr6/A12	390	44.0366	9.92
	*GrWRI2-likea*	Cotton_D_gene_10014405	Chr6/D09	282	32.2474	9.85
	*GrWRI2-likeb*	Cotton_D_gene_10014404	Chr6/D09	387	44.1780	8.89
	*GrWRI2-likec*	Cotton_D_gene_10008870	Chr8/D12	387	43.5171	9.84
	*GrWRI2-liked*	Cotton_D_gene_10002133	scaffold384	368	41.8326	9.51
	*GrWRI2-likee*	Cotton_D_gene_10001570	scaffold484	386	44.0276	8.52

**FIGURE 1:**
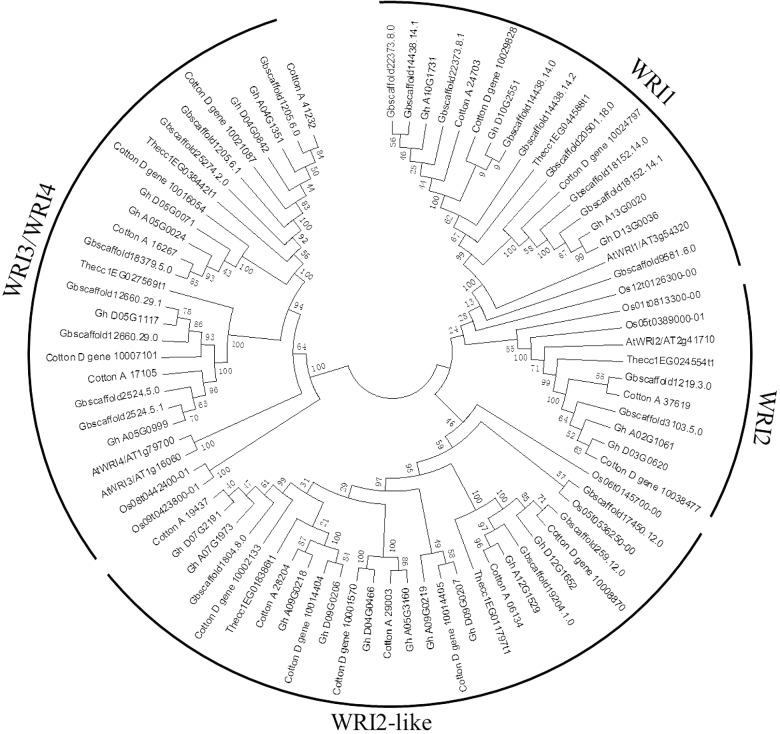
Phylogenetic tree of *WRI-like* gene family in *Gossypium* and *Arabidopsis*. The phylogenetic tree of all WRI-like proteins in four *Gossypium* species and *Arabidopsis* was constructed using the neighbor-joining method with 1,000 bootstrap replicates.

A phylogenetic tree was constructed to reveal the relationships of WRI-like proteins in *Arabidopsis*, cacao, rice, and cotton (**Figure 1**). This phylogenetic analysis classified *WRI-like* genes into *WRI1*, *WRI2*, *WRI3*/*WRI4*, and *WRI2-like* subfamilies. In comparison with dicotyledonous *Arabidopsis*, a more *WRI2-like* subfamily was identified interestingly. The *WRI1* subfamily contained 11 members: 4 from *G. hirsutum*, 4 from *G. barbadense*, 1 from *G. arboreum*, and 2 from *G. raimondii*. The *WRI2* subfamily consisted of seven members: two from *G. hirsutum*, three from *G. barbadense*, and one each from *G. arboreum* and *G. raimondii*. The *WRI3*/*WRI4* subfamily included 18 members: 6, 6, 3, and 3 from *G. hirsutum*, *G. barbadense*, *G. arboreum*, and *G. raimondii*, respectively. Finally, the *WRI2-like* subfamily comprised 23 members: 10, 4, 4, and 5 in *G. hirsutum*, *G. barbadense*, *G. arboreum*, and *G. raimondii*, respectively.

### Chromosomal Distribution of *WRI-Like* Genes

**FIGURE 2:**
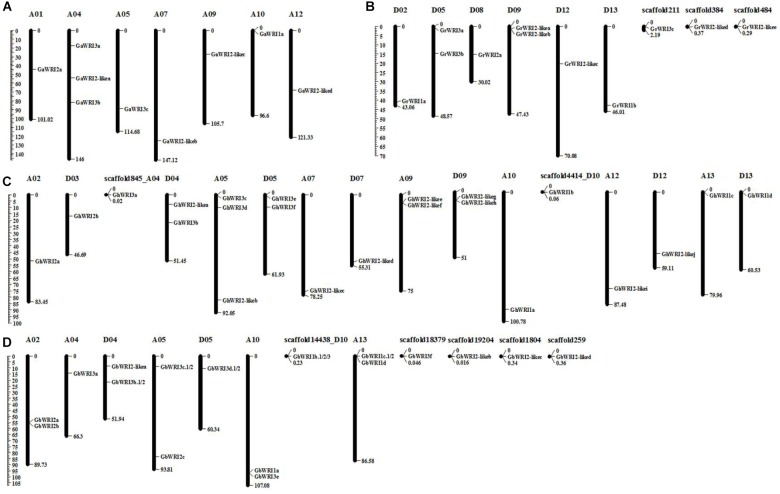
Chromosomal localization of *WRI-like* genes in four *Gossypium* species. A total of 69 *WRI-like* genes were mapped onto different chromosomes or scaffolds of *G. arboreum* **(A)**, *G. raimondii* **(B)**, *G. hirsutum* **(C)**, and *G. barbadense* **(D)**. The scale represents the megabases (Mb).

The identified *WRI-like* genes were physically mapped to the chromosomes of cotton using the reference genome sequences (**Figure 2** and **Table 1**). In the *G. arboretum* genome, nine *GaWRIs* were evenly distributed on seven chromosomes (A01, A04, A05, A07, A09, A10, and A12) (**Figure 2A**). One *GaWRI* gene each was located on chromosomes A01, A05, A07, A09, A10, and A12, and three *GaWRI* genes were found on chromosome A04. Nine of the 11 *GrWRI* genes in the *G. raimondii* genome were uniformly distributed on six chromosomes (D02, D05, D08, D09, D12, and D13), with one each positioned on chromosomes D05 and D09 (**Figure 2B**). The other three *GrWRI* genes were only located on the scaffolds. Among the 22 *GhWRI* genes identified in the *G. hirsutum* genome, 11 originated from the eight At subgenome chromosomes (A02, A04, A05, A07, A09, A10, A12, and A13), while 11 were derived from the eight Dt subgenome chromosomes (D03, D04, D05, D07, D09, D10, D12, and D13) (**Figure 2C**). Two genes each were located on chromosomes D04, D05, A09, and D09, while chromosome A05 harbored three *GhWRIs*. Each of the remaining chromosomes contained one *GhWRI* gene each. Among the 17 *GbWRI* genes identified in the *G. barbadense* genome, nine were located on the five At subgenome chromosomes (two on A02, one on A04, two on A05, two on A10, and two on A13), four were mapped to the three Dt subgenome chromosomes (two on D04, one on D05, and one on D10), and four were located on scaffolds (**Figure 2D**). Most of *WRI-like* genes were distributed evenly on the chromosomes (**Figure 2** and **Table 1**), which provided a clue to their evolution.

### Analysis of Gene Duplication and Loss of *WRI-Like* Genes

Large-scale duplication events have occurred during *Gossypium* evolution progress (Paterson et al., 2012). Gene duplication events, including tandem and segmental duplications, are considered as the major forces for expansion of gene families. In contrast to *Arabidopsis*, cacao, and rice, the *WRI-like* genes were expanded in cotton (**Figure 1**). We investigated the possible tandem and segmental duplication events of *WRI-like* genes in the four cotton species, respectively (**Table 2** and **Supplementary Table S2**). Among them, no duplicated gene pairs were found in genome of *G. raimondii* and *G. arboretum*. In *G. hirsutum*, nine duplicated gene pairs were found to be segmental duplication events. In *G. barbadense*, six duplicated gene pairs were found, containing five segmental duplication events and one tandem event. These results indicated that segmental duplication were the main driving forces of the in the expansion of the *WRI-like* gene family.

**Table 2:** *K*a and *K*s calculations of the *WRI-like* duplicated gene pairs.

Species	*Gene1*	*Gene2*	*K*a	*K*s	*K*a/*K*s	Duplicated type
*G. hirsutum*	*GhWRI1a*	*GhWRI1b*	0.0284	0.0754	0.376658	Segmental duplication
	*GhWRI2a*	*GhWRI2b*	0.9995	0.9121	1.095823	Segmental duplication
	*GhWRI3a*	*GhWRI3b*	1.5293	1.5128	1.010907	Segmental duplication
	*GhWRI3c*	*GhWRI3e*	0.0126	0.0613	0.205546	Segmental duplication
	*GhWRI3d*	*GhWRI3f*	0.0099	0.0436	0.227064	Segmental duplication
	*GhWRI2-likea*	*GhWRI2-likeb*	2.5991	2.3147	1.122867	Segmental duplication
	*GhWRI2-likec*	*GhWRI2-liked*	0.8933	0.7883	1.140432	Segmental duplication
	*GhWRI2-likee*	*GhWRI2-likeg*	0.0135	0.0350	0.385714	Segmental duplication
	*GhWRI2-likei*	*GhWRI2-likej*	0.1420	0.1502	0.945406	Segmental duplication
*G. barbadense*	*GbWRI1a*	*GbWRI1b.1*	0.0251	0.0636	0.394654	Segmental duplication
	*GbWRI2a*	*GbWRI2b*	0.0052	0.0102	0.509804	Tandem duplication
	*GbWRI3a*	*GbWRI3b.1*	0.0180	0.0248	0.725806	Segmental duplication
	*GbWRI3c.1*	*GbWRI3d.1*	0.0098	0.0387	0.253230	Segmental duplication
	*GbWRI2-likea*	*GbWRI2c*	0.0166	0.0300	0.553333	Segmental duplication
	*GbWRI2-likeb*	*GbWRI2-liked*	0.1318	0.1312	1.004573	Segmental duplication

During the process of evolution, gene pairs are subject to three alternative fates, i.e., non-functionalization, subfunctionalization, and neofunctionalization (Lynch and Conery, 2000). In this study, the *K*a/*K*s ratios for 15 duplicated *WRI-like* gene pairs were calculated (**Table 2**). The *K*a/*K*s ratios of ten pairs were less than 1, which suggests that these duplicated *WRI-like* genes have mainly experienced purifying selection pressure. The *K*a/*K*s ratios of other five pairs were more than 1, indicating positive selection pressure in the progress of evolution.

**FIGURE 3:**
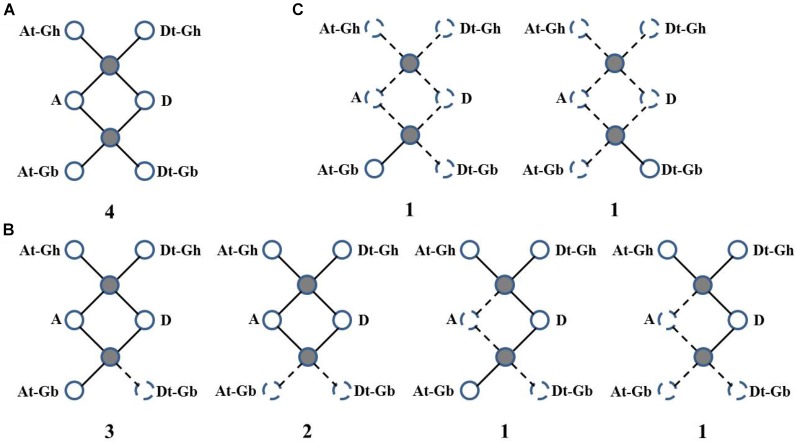
Gene conservation and loss analysis of the *WRI-like* genes in *Gossypium*. **(A)** Ultra-conserved homologous *WRI-like* clusters in four cotton species. **(B)** Homologous *WRI-like* genes lost from one and/or two cotton species. **(C)** *WRI-like* genes only present in *G. barbadense*. Statistics and scenarios of gene conservation and loss analysis in the four cotton species. The observed genes were indicated as solid lines and balls, and the lost genes were indicated as dotted lines and balls. The number of gene clusters found in the four cotton species is provided below each graphic.

Then, *WRI-like* gene conservation and loss were analyzed based on the best match and the syntenic blocks in the CottonGen website (**Figure 3** and **Supplementary Table S3**). Four homologous *WRI-like* clusters were ultra-conserved in four cotton species (**Figure 3A** and **Supplementary Table S3**). Ten homologous *WRI-like* genes were lost from the At, Dt or both subgenomes of G. *barbadense* and two were lost from *G. arboretum* (**Figure 3B** and **Supplementary Table S3**). Additionally, two genes were only present in *G. barbadense* (**Figure 3C** and **Supplementary Table S3**). This indicated that the *GbWRIs* and *GaWRIs* experienced a higher frequency of genic sequence losses than *GhWRIs* and *GrWRIs*.

### Gene Structure and Protein Domain Analyses of WRI-Likes in *G. hirsutum*

**FIGURE 4:**
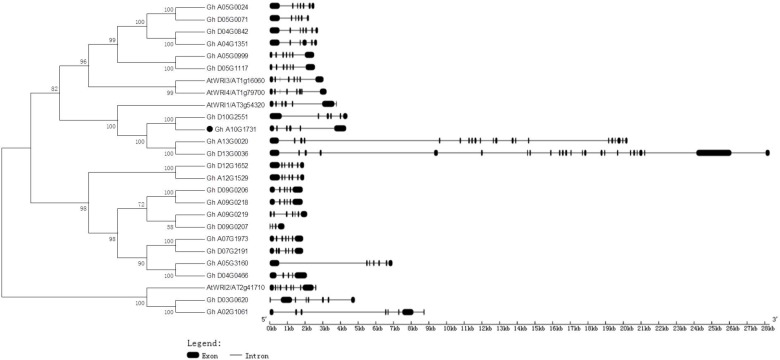
Phylogenetic relationships and gene structures of GhWRIs. Phylogeny of 22 GhWRI proteins in *G. hirsutum* and *Arabidopsis*. The phylogenetic tree **(Left)** was constructed using the neighbor-joining (NJ) method with 1,000 bootstrap replicates. The exon–intron structure **(Right)** of *GhWRI* genes was drawn using the GSDS server. Black boxes show exons, and lines show introns. The scale bar is shown at the bottom.

Generic Feature Format files of the four *Gossypium* species and a phylogenetic tree of deduced amino acids of GhWRIs were used to analyze the similarity and diversity of their exon–intron structures (**Figure 4**). The *AtWRI2* gene contained 10 introns and 11 exons, whereas *WRI2* subfamily genes *GhWRI2a* (Gh_A02G1061) and *GhWRI2b* (Gh_D03G0620) harbored seven introns and eight exons. AtWRI1, AtWRI3, and AtWRI4 genes contained seven introns and eight exons. In contrast, most *GhWRI1*, *GhWRI3/GhWRI4*, and *GhWRI2-like* family genes fell into two categories: those containing five introns and six exons, and those having six introns and seven exons. *GhWRI3b* (Gh_D04G0842), *GhWRI3c* (Gh_A05G0024), *GhWRI3d* (Gh_A05G0999), *GhWRI3f* (Gh_D05G1117), *GhWRI2-likeb* (Gh_A05G3160), *GhWRI2-likec* (Gh_A07G1973), *GhWRI2-liked* (Gh_D07G2191), *GhWRI2-likef* (Gh_A09G0219), *GhWRI2-likei* (Gh_A12G1529), and *GhWRI2-likej* (Gh_D12G1652) contained six introns and seven exons. *GhWRI1a* (Gh_A10G1731), *GhWRI1b* (Gh_D10G2551), *GhWRI3a* (Gh_A04G1351), *GhWRI3e* (Gh_D05G0071), *GhWRI2-likee* (Gh_A09G0218), and *GhWRI2-likeg* (Gh_D09G0206) contained five introns and six exons. Four genes had unique intron–exon compositions: *GhWRI1c* (Gh_A13G0020) with 20 introns and 21 exons, *GhWRI1d* (Gh_D13G0036) with 24 introns and 25 exons, *GhWRI2-likea* (Gh_D04G0466) with four introns and five exons, and *GhWRI2-likeh* (Gh_D09G0207) containing three introns and four exons.

**FIGURE 5:**
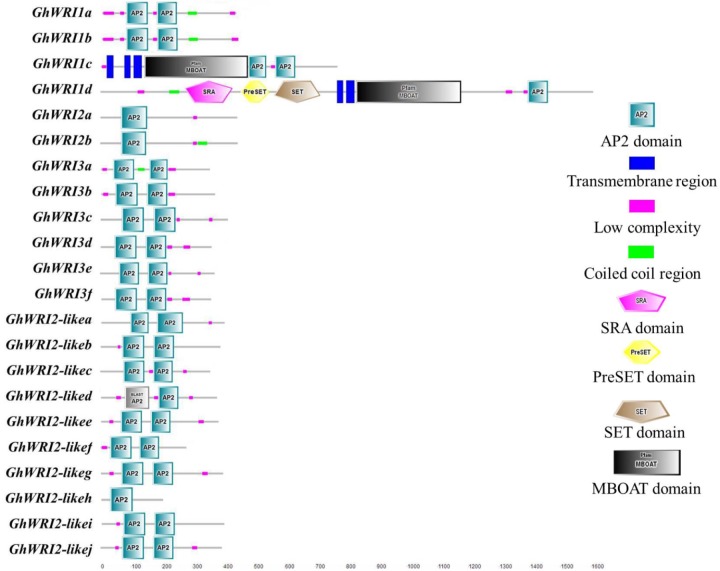
GhWRI protein domain prediction. Potential AP2 domains of GhWRI proteins were identified using the SMART database.

To better understand the similarity and diversity of GhWRI protein structures, their putative protein domains were predicted using the SMART database. The WRI-like proteins belonged to the AP2-EREPB family of transcription factors (Cernac and Benning, 2004; To et al., 2012). As shown in **Figure 5**, most GhWRIs contained two AP2 domains; the exceptions were GhWRI1d, GhWRI2a, GhWRI2b, and GhWRI2-likeh, all having only one each. Interestingly, we also found many other putative protein domains in *GhWRI1c* and *GhWRI1d* that need to be further verified.

### Tissue-Specific Expression Profiles of *GhWRI* Genes

**FIGURE 6:**
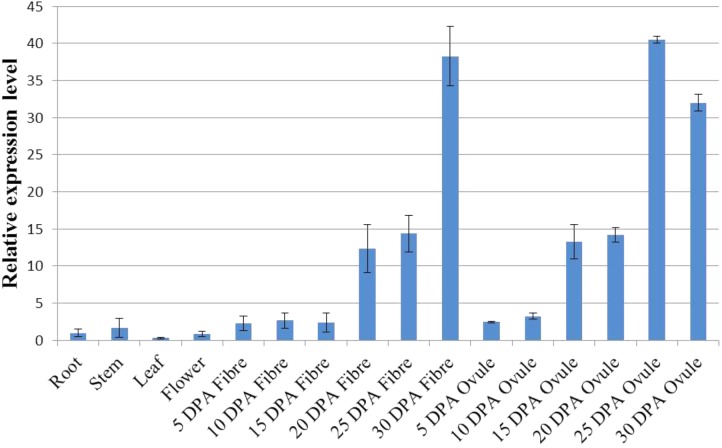
Tissue-specific expression profile of *GhWRI1a* in different tissues of *G. hirsutum* accession TM-1. The ΔC_t_ value of *GhWRI1a* in root was set as the control. The data presented are the means ± SD of three replicates.

The expression pattern of a gene can be a direct indication of its involvement in developmental or differential events (Zang et al., 2017). To reveal the tissue-specific expression profiles of the 22 *GhWRI* genes identified in this study, published TM-1 expression data (Zhang et al., 2015) were used to analyze the transcript profiles of *GhWRI* genes in 22 cotton tissues (**Supplementary Figure S2**). *GhWRI* genes from *WRI1*, *WRI2*, and *WRI3/WRI4* subfamilies were widely detected in different tissues, whereas *GhWRI* genes from the *WRI2-like* subfamily exhibited very low expression levels in most tissues. Interestingly, we found *GhWRI1a* and *GhWRI1b* (gene pairs from the corresponding At and Dt subgenome) were highly expressed in 20–35 DPA ovules (**Figure 6** and **Supplementary Figure S2**). *GhWRI1a* was thus selected for further functional analysis.

### Ectopic Expression of *GhWRI1a* Rescued the Seed Phenotype of the *wri1-7* Mutant and Increased the Oil Content of *Arabidopsis* Seeds

**FIGURE 7:**
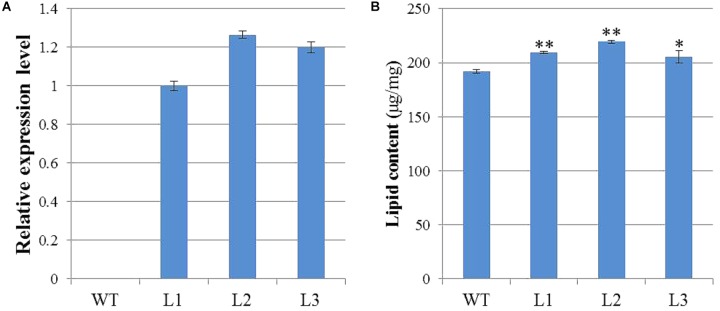
Improved oil content of *GhWRI1a* transgenic plants. **(A)** Relative expression level of *GhWRI1a* in three transgenic *Arabidopsis* lines (L1, L2, and L3). The ΔC_t_ value of *GhWRI1a* in transgenic line L1 was set as the control. The data presented are the means ± SD of three replicates. **(B)** Seed oil content of *GhWRI1a* transgenic lines (L1, L2, and L3) and WT. The data presented are the means ± SD of three replicates; ^∗^*P* < 0.05; ^∗∗^*P* < 0.01 (Student’s *t*-test).

To characterize the biological functions of *GhWRI1a* in regard to oil content, we generated transgenic *Arabidopsis* plants overexpressing *GhWRI1a*. qRT-PCR was performed to analyze relative expression levels of *GhWRI1a* in transgenic *Arabidopsis* using cDNA from three different transgenic lines and WT as templates (**Figure 7A**). *GhWRI1a* was highly expressed in the transgenic lines. To evaluate the applicability of *GhWRI1a* in transgenic breeding for oil content, we characterized the phenotypes of *GhWRI1a* transgenic *Arabidopsis* at different developmental stages. No visible difference between transgenic and WT plants was observed (data not shown). To determine whether *GhWRI1a* had increased the oil content, we compared the oil contents of transgenic and WT plants. Significantly increased oil content, 6.96–14.24% higher, was observed in the transgenic plants (**Figure 7B**).

**FIGURE 8:**
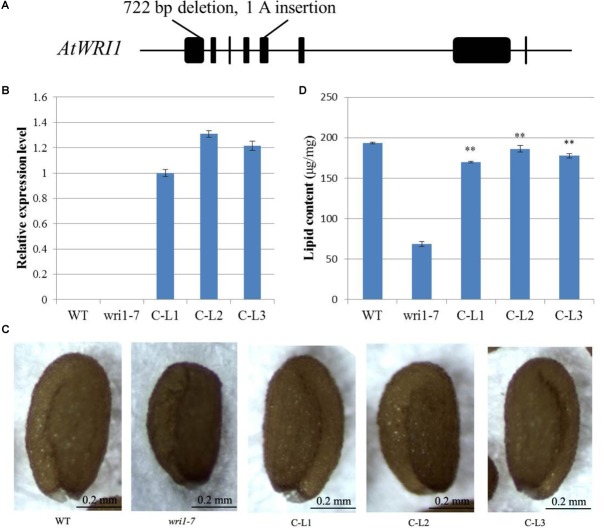
*Arabidopsis wri1-7* mutant displaying a wrinkled phenotype rescued by overexpression of *GhWRI1a*. **(A)** Isolation and molecular characterization of the *wri1-7* mutant. **(B)** Relative expression level of *GhWRI1a* in three *GhWRI1a*-complemented lines (C-L1, C-L2, and C-L3). The ΔC_t_ value of *GhWRI1a* in complemented line C-L1 was set as the control. The data presented are the means ± SD of three replicates. **(C)** Rescue of the wrinkled phenotype of mature *wri1-4* seeds by overexpression of *GhWRI1a*. Dry seeds from complemented lines were observed by stereoscopic microscopy. Representative seeds are shown. Bar = 0.2 mm. **(D)** Seed oil content of complemented lines (C-L1, C-L2, and C-L3), *wri1-7*, and WT. The data presented are the means ± SD of three replicates; ^∗∗^*P* < 0.01 (Student’s *t*-test).

In order to determine whether the *GhWRI1a* transcription factor is involved in the activation of the whole fatty acid biosynthetic pathway, we created an *atwri1* mutant named *wri1-7* by the CRISPR method. DNA sequence comparison revealed the presence of a 722 bp deletion and a single adenine (A) insertion from the first to the fifth exon in the *wri1-7* mutant (**Figure 8A** and **Supplementary Figure S1**). Microscopic observation of mature dry seeds of the *wri1-7* mutant also revealed a wrinkled phenotype (**Figure 8B**), similar to previously reported *wri1* mutant seeds (Cernac and Benning, 2004; To et al., 2012). The ability of the overexpression constructs to complement the seed phenotype of the *wri1-7* mutant was confirmed by crossing L1, L2, and L3 transgenic plants with the *wri1-7* mutant. Over accumulation of *GhWRI1a* RNA in the transgenic lines was verified by qRT-PCR (**Figure 8B**). Microscopic observation of mature dry seeds revealed a reversion to the wrinkled phenotype in *wri1-7* seeds overexpressing *GhWRI1a* (**Figure 8C**). An analysis of total oil content of the dry seeds confirmed the ability of *GhWRI1a* to efficiently activate fatty acid biosynthesis and to thus complement the oil accumulation of *wri1-7* seeds (**Figure 8D**).

## Discussion

Numerous studies have revealed a crucial role for *WRI-like* genes in TAG biosynthesis, including *GhWRI1* corresponding to *GhWRI1b* (Liu et al., 2018). Nevertheless, the naming of *WRI-like* family genes in cotton is confusing, and their systematic exposition is incomplete. In this study, we have accomplished the first-ever identification of *WRI-like* genes in four representative types of cotton, i.e., allotetraploid cotton species *G. hirsutum* and *G. barbadense* and their diploid ancestors *G. arboreum*, and *G. raimondii*. Our findings provide significant insights into the sequence variation, adaptive evolution, protein domains, expression profiles, co-localization with QTLs and *GhWRI1a* functions in cotton.

Our analysis revealed details of 22 deduced GhWRIs, most of which contain two AP2 domains, with only four GhWRIs (GhWRI1d, GhWRI2a, GhWRI2b, and GhWRI2-likeh) having just one AP2 domain (**Figure 5**). The *WRI-like* gene family is a branch of the AP2/EREBP (APETALA2/ethylene responsive element binding protein) transcription factor family. The AP2/EREBP family is one of the largest plant transcription factor families and plays an important role in plant growth and development (Okamuro et al., 1997; Riechmann et al., 2000; Zhou et al., 2013). This superfamily, comprising AP2, EREBP, and RAV subfamilies, is defined by the AP2/ERF DNA binding domain. AP2 family proteins contain two repeated AP2/ERF domains, EREBP family proteins have a single AP2/ERF domain, and RAV family proteins possess a B3 DNA-binding domain in addition to a single AP2/ERF domain (Sakuma et al., 2002; Feng et al., 2005; Nakano et al., 2006). AtWRI1, AtWRI2, AtWRI3, and AtWRI4 proteins all belong to the AP2 subfamily (Feng et al., 2005). These proteins generally contain two repeated AP2/ERF domains, the exception is AtWRI2, which was found to possess only one AP2/ERF domain (**Supplementary Figure S3**), consistent with previous reports (Nakano et al., 2006). Consequently, GhWRI1d, GhWRI2a, GhWRI2b, and GhWRI2-likeh, which contain only one AP2 domain, are typical representatives of the AP2 subfamily. The *WRI-like* genes identified in this study belong to the AP2 subfamily of the AP2/EREBP family.

Cottonseed oil accumulates in ovules after 15 DPA. At this stage, most *GhWRIs* were found to be expressed in our study. *GhWRI1a* and *GhWRI1b* had the highest expression levels (**Supplementary Figure S2**), indicating that these two genes play important roles in TAG biosynthesis in developing cotton seeds. In this study, we demonstrated that ectopic expression of *GhWRI1a* could rescue the seed phenotype of the *wri1-7* mutant and increase the oil content of *Arabidopsis* seeds. In addition, four *WRI-like* genes were localized in cottonseed oil QTL intervals, which suggests their association with natural variation in cottonseed oil content.

We further discovered that *GhWRIs* were expressed in developing fibers. *GhWRI1a* and *GhWRI1b*, in particular, were highly expressed in 25-DPA developing fibers (**Figure 6** and **Supplementary Figure S2**), suggesting their additional involvement in fiber development. Other studies of upland cotton have also indicated the involvement of *GhWRIs* in fiber length (Qu et al., 2012; Qaisar et al., 2017). The regulatory relationship between *GhWRIs* and fiber development needs to be further verified.

In short, we have performed a comprehensive genome-wide analysis of the *WRI-like* gene family in *G. hirsutum*, *G. barbadense*, *G. raimondii*, and *G. arboreum*. A total of 69 *WRI-like* genes grouped into four distinct subfamilies were identified in four sequenced *Gossypium* species. Our detailed analysis has established a solid foundation for further studies of *WRI-like* genes in cotton.

## Author Contributions

JY and XZ directed the experiments. WP, MW, YG, NW, GL, JM, DL, YC, XL, and JZ participated in the study. XZ conceived the study, performed the experiments and wrote the manuscript. JY and JZ revised the manuscript. All authors read and approved the final manuscript.

## Conflict of Interest Statement

The authors declare that the research was conducted in the absence of any commercial or financial relationships that could be construed as a potential conflict of interest.

## Footnotes

1 https://www.cottongen.org/

2 http://www.arabidopsis.org/

3 https://www.cacaogenomedb.org/

4 https://rapdb.dna.affrc.go.jp/

5 http://web.expasy.org/

6 http://www.earthatlas.mapchart.com/

7 http://www.cottonqtldb.org/

8 http://gsds.cbi.pku.edu.cn/

9 http://smart.embl-heidelberg.de/

## Supplementary Material

FIGURE S1 Sequence alignment of *AtWRI1* in the *wri1-7* mutant and WT. Target1 and Target2 are the two designed single-guide RNAs.

FIGURE S2 Expression analysis of *GhWRIs* in 22 tissues of *G. hirsutum* accession TM-1 (Zhang et al., 2015). The RNA-seq profiles of TM-1 were used to identify *GhWRI* gene expression levels. FPKM, fragments per kilobase of exon model per million mapped reads.

FIGURE S3 Protein domain prediction for AtWRIs. The potential AP2 domains of AtWRI proteins were identified using the SMART database.

TABLE S1 Primers used in this paper.

TABLE S2 Duplicated genes of *WRI-like* family in *Gossypium*.

TABLE S3 Gene conservation and loss analysis of *WRI-like* family in *Gossypium*.

FILE S1 Phylogenetic data of **Figure 1**.

FILE S2 Coding sequence of *GhWRI1a*.

## References

[B1] An D. Suh M. C. (2015). Overexpression of *Arabidopsis* WRI1 enhanced seed mass and storage oil content in *Camelina sativa*. *Plant Biotechnol. Rep.* 9 137–148. 10.1007/s11816-015-0351-x

[B2] An D. Kim H. Ju S. Go Y. S. Kim H. U. Suh M. C. (2017). Expression of *Camelina* WRINKLED1 isoforms rescue the seed phenotype of the *Arabidopsis wri1* mutant and increase the triacylglycerol content in tobacco leaves. *Front. Plant Sci.* 8:34. 10.3389/fpls.2017.00034 28174580 PMC5258696

[B3] Bates P. D. Browse J. (2012). The significance of different diacylgycerol synthesis pathways on plant oil composition and bioengineering. *Front. Plant Sci.* 3:147. 10.3389/Fpls.2012.00147 22783267 PMC3387579

[B4] Cernac A. Benning C. (2004). WRINKLED1 encodes an AP2/EREB domain protein involved in the control of storage compound biosynthesis in Arabidopsis. *Plant J.* 40 575–585. 10.1111/j.1365-313X.2004.02235.x 15500472

[B5] Cui Y. P. Liu Z. J. Zhao Y. P. Wang Y. M. Huang Y. Li L. (2017). Overexpression of heteromeric GhACCase subunits enhanced oil accumulation in upland cotton. *Plant Mol. Biol. Rep.* 35 287–297. 10.1007/s11105-016-1022-y

[B6] Dong Y. Li C. Zhang Y. He Q. Daud M. K. Chen J. (2016). Glutathione S-transferase gene family in *Gossypium raimondii* and *G. arboreum*: comparative genomic study and their expression under salt stress. *Front. Plant Sci.* 7:139. 10.3389/Fpls.2016.00139 26904090 PMC4751282

[B7] Feng J. X. Liu D. Pan Y. Gong W. Ma L. G. Luo J. C. (2005). An annotation update via cDNA sequence analysis and comprehensive profiling of developmental, hormonal or environmental responsiveness of the Arabidopsis AP2/EREBP transcription factor gene family. *Plant Mol. Biol.* 59 853–868. 10.1007/s11103-005-1511-0 16307362

[B8] Focks N. Benning C. (1998). wrinkled1: a novel, low-seed-oil mutant of Arabidopsis with a deficiency in the seed-specific regulation of carbohydrate metabolism. *Plant Physiol.* 118 91–101. 10.1104/pp.118.1.91 9733529 PMC34877

[B9] Grimberg A. Carlsson A. S. Marttila S. Bhalerao R. Hofvander P. (2015). Transcriptional transitions in *Nicotiana benthamiana* leaves upon induction of oil synthesis by WRINKLED1 homologs from diverse species and tissues. *BMC Plant Biol.* 15:192. 10.1186/s12870-015-0579-1 26253704 PMC4528408

[B10] Hofvander P. Ischebeck T. Turesson H. Kushwaha S. K. Feussner I. Carlsson A. S. (2016). Potato tuber expression of Arabidopsis WRINKLED1 increase triacylglycerol and membrane lipids while affecting central carbohydrate metabolism. *Plant Biotechnol. J.* 14 1883–1898. 10.1111/pbi.12550 26914183 PMC5069604

[B11] Larkin M. A. Blackshields G. Brown N. P. Chenna R. McGettigan P. A. McWilliam H. (2007). Clustal W and clustal X version 2.0. *Bioinformatics* 23 2947–2948. 10.1093/bioinformatics/btm404 17846036

[B12] Li F. Fan G. Wang K. Sun F. Yuan Y. Song G. (2014). Genome sequence of the cultivated cotton Gossypium arboreum. *Nat. Genet.* 46 567–572. 10.1038/ng.2987 24836287

[B13] Liu Q. Singh S. P. Green A. G. (2002). High-stearic and high-oleic cottonseed oils produced by hairpin RNA-mediated post-transcriptional gene silencing. *Plant Physiol.* 129 1732–1743. 10.1104/pp.001933 12177486 PMC166761

[B14] Liu Q. Surinder S. Chapman K. Green A. (2009). “Bridging traditional and molecular genetics in modifying cottonseed oil,” in *Genetics and Genomics of Cotton. Plant Genetics and Genomics: Crops and Models*, Vol. 3 ed. Paterson A. H. (London: Springer Science + Business Media),353–382.

[B15] Liu Z. J. Zhao Y. P. Liang W. Cui Y. P. Wang Y. M. Hua J. P. (2018). Over-expression of transcription factor GhWRI1 in upland cotton. *Biol. Plantarum.* 62 335–342. 10.1007/s10535-018-0777-4

[B16] Liu J. Hua W. Zhan G. Wei F. Wang X. Liu G. (2010). Increasing seed mass and oil content in transgenic *Arabidopsis* by the overexpression of wri1-like gene from *Brassica napus*. *Plant Physiol. Biochem.* 48 9–15. 10.1016/j.plaphy.2009.09.007 19828328

[B17] Livak K. J. Schmittgen T. D. (2001). Analysis of relative gene expression data using real-time quantitative PCR and the 2(T)(-Delta Delta C) method. *Methods* 25 402–408. 10.1006/meth.2001.1262 11846609

[B18] Lynch M. Conery J. S. (2000). The evolutionary fate and consequences of duplicate genes. *Science* 290 1151–1155. 10.1126/science.290.5494.1151 11073452

[B19] Nakano T. Suzuki K. Fujimura T. Shinshi H. (2006). Genome-wide analysis of the ERF gene family in Arabidopsis and rice. *Plant Physiol.* 140 411–432. 10.1104/pp.105.073783 16407444 PMC1361313

[B20] Okamuro J. K. Caster B. Villarroel R. Van Montagu M. Jofuku K. D. (1997). The AP2 domain of APETALA2 defines a large new family of DNA binding proteins in *Arabidopsis*. *Proc. Natl. Acad. Sci. U.S.A.* 94 7076–7081. 10.1073/pnas.94.13.7076 9192694 PMC21287

[B21] Paterson A. H. Wendel J. F. Gundlach H. Guo H. Jenkins J. Jin D. (2012). Repeated polyploidization of *Gossypium* genomes and the evolution of spinnable cotton fibres. *Nature* 492 423–427. 10.1038/nature11798 23257886

[B22] Pouvreau B. Baud S. Vernoud V. Morin V. Py C. Gendrot G. (2011). Duplicate maize Wrinkled1 transcription factors activate target genes involved in seed oil biosynthesis. *Plant Physiol.* 156 674–686. 10.1104/pp.111.173641 21474435 PMC3177267

[B23] Qaisar U. Akhtar F. Azeem M. Yousaf S. (2017). Studies on involvement of Wrinkled1 transcription factor in the development of extra-long staple in cotton. *Indian J. Genet. Plant Breed.* 77 298–303. 10.5958/0975-6906.2017.00040.2

[B24] Qu J. Ye J. Geng Y. F. Sun Y. W. Gao S. Q. Zhang B. P. (2012). Dissecting functions of KATANIN and WRINKLED1 in cotton fiber development by virus-induced gene silencing. *Plant Physiol.* 160 738–748. 10.1104/pp.112.198564 22837356 PMC3461552

[B25] Riechmann J. L. Heard J. Martin G. Reuber L. Jiang C. Keddie J. (2000). Arabidopsis transcription factors: genome-wide comparative analysis among eukaryotes. *Science* 290 2105–2110. 10.1126/science.290.5499.2105 11118137

[B26] Sakuma Y. Liu Q. Dubouzet J. G. Abe H. Shinozaki K. Yamaguchi-Shinozaki K. (2002). DNA-binding specificity of the ERF/AP2 domain of *Arabidopsis* DREBs, transcription factors involved in dehydration- and cold-inducible gene expression. *Biochem. Biophys. Res. Commun.* 290 998–1009. 10.1006/bbrc.2001.6299 11798174

[B27] Shen B. Allen W. B. Zheng P. Li C. Glassman K. Ranch J. (2010). Expression of ZmLEC1 and ZmWRI1 increases seed oil production in maize. *Plant Physiol.* 153 980–987. 10.1104/pp.110.157537 20488892 PMC2899924

[B28] Tamura K. Stecher G. Peterson D. Filipski A. Kumar S. (2013). MEGA6: molecular evolutionary genetics analysis version 6.0. *Mol. Biol. Evol.* 30 2725–2729. 10.1093/molbev/mst197 24132122 PMC3840312

[B29] To A. Joubès J. Barthole G. Lécureuil A. Scagnelli A. Jasinski S. (2012). WRINKLED transcription factors orchestrate tissue-specific regulation of fatty acid biosynthesis in *Arabidopsis*. *Plant Cell* 24 5007–5023. 10.1105/tpc.112.106120 23243127 PMC3556972

[B30] Voorrips R. E. (2002). MapChart: software for the graphical presentation of linkage maps and QTLs. *J. Hered.* 93 77–78. 10.1093/jhered/93.1.77 12011185

[B31] Wang P. Zhang J. Sun L. Ma Y. Xu J. Liang S. (2017). High efficient multi-sites genome editing in allotetraploid cotton (*Gossypium hirsutum*) using CRISPR/Cas9 system. *Plant Biotechnol. J.* 16 137–150. 10.1111/pbi.12755 28499063 PMC5785356

[B32] Wang K. Wang Z. Li F. Ye W. Wang J. Song G. (2012). The draft genome of a diploid cotton *Gossypium raimondii*. *Nat. Genet.* 44 1098–1103. 10.1038/ng.2371 22922876

[B33] Yang Y. Munz J. Cass C. Zienkiewicz A. Kong Q. Ma W. (2015). Ectopic expression of WRINKLED1 affects fatty acid homeostasis in *Brachypodium distachyon* vegetative tissues. *Plant Physiol.* 169 1836–1847. 10.1104/pp.15.01236 26419778 PMC4634098

[B34] Yuan D. Tang Z. Wang M. Gao W. Tu L. Jin X. (2015). The genome sequence of Sea-Island cotton (*Gossypium barbadense*) provides insights into the allopolyploidization and development of superior spinnable fibres. *Sci. Rep.* 5:17662. 10.1038/srep17662 26634818 PMC4669482

[B35] Zang X. Geng X. Liu K. Wang F. Liu Z. Zhang L. (2017). Ectopic expression of TaOEP16-2-5B, a wheat plastid outer envelope protein gene, enhances heat and drought stress tolerance in transgenic *Arabidopsis* plants. *Plant Sci.* 258 1–11. 10.1016/j.plantsci.2017.01.011 28330552

[B36] Zhang T. Hu Y. Jiang W. Fang L. Guan X. Chen J. (2015). Sequencing of allotetraploid cotton (*Gossypium hirsutum* L. acc. TM-1) provides a resource for fiber improvement. *Nat. Biotechnol.* 33 531–537. 10.1038/nbt.3207 25893781

[B37] Zhang Y. He P. Yang Z. Huang G. Wang L. Pang C. (2017). A genome-scale analysis of the PIN gene family reveals its functions in cotton fiber development. *Front. Plant Sci.* 8:461. 10.3389/fpls.2017.00461 28424725 PMC5371604

[B38] Zhou Y. Xia H. Li X. J. Hu R. Chen Y. Li X. B. (2013). Overexpression of a cotton gene that encodes a putative transcription factor of AP2/EREBP family in *Arabidopsis* affects growth and development of transgenic plants. *PLoS One* 8:e78635. 10.1371/journal.pone.0078635 24194949 PMC3806861

