# Supplementary figures and images for: Genome-Scale Analysis of the WRI-Like Family in Gossypium and Functional Characterization of GhWRI1a Controlling Triacylglycerol Content
Source: Front Plant Sci. 2018 Oct 16;9:1516. doi: 10.3389/fpls.2018.01516 (PMC6198791; doi:10.3389/fpls.2018.01516)

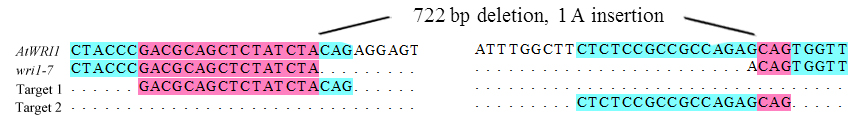

Supplement: FIGURE S1 — Sequence alignment of AtWRI1 in the wri1-7 mutant and WT. Target1 and Target2 are the two designed single-guide RNAs. [file Image_1.JPEG]

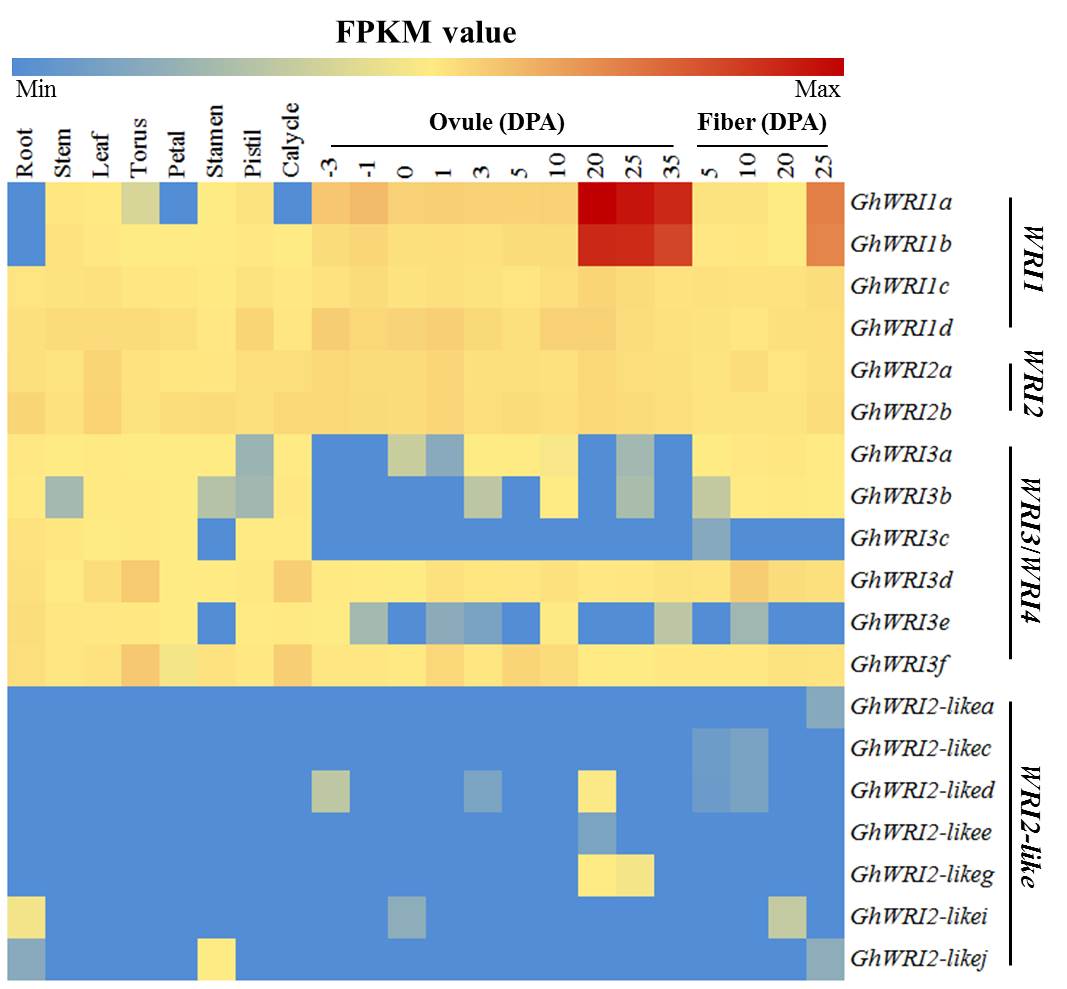

Supplement: FIGURE S2 — Expression analysis of GhWRIs in 22 tissues of G. hirsutum accession TM-1 (Zhang et al., 2015). The RNA-seq profiles of TM-1 were used to identify GhWRI gene expression levels. FPKM, fragments per kilobase of exon model per million mapped reads. [file Image_2.JPEG]

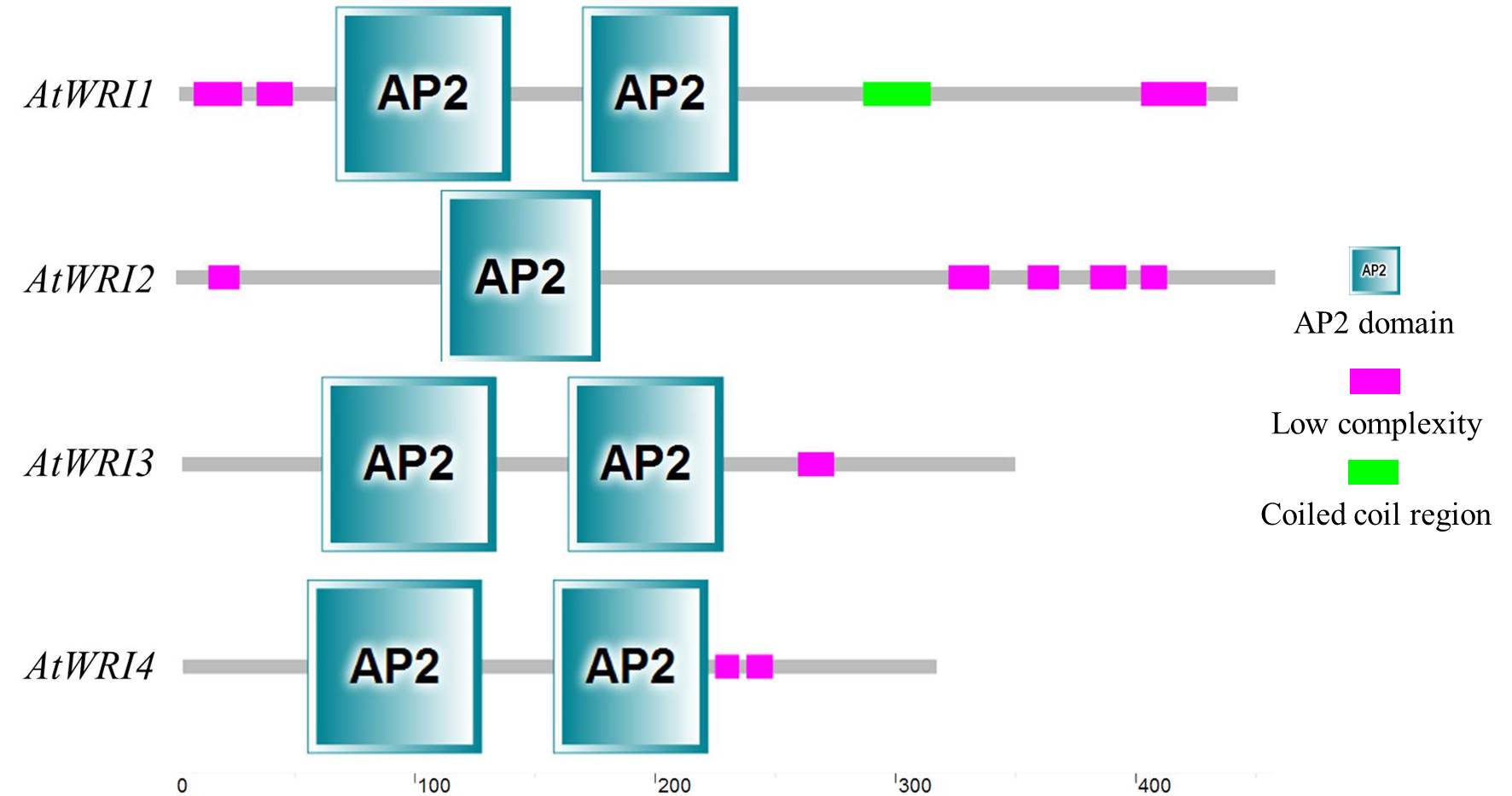

Supplement: FIGURE S3 — Protein domain prediction for AtWRIs. The potential AP2 domains of AtWRI proteins were identified using the SMART database. [file Image_3.JPEG]
