# Supplementary material for: Genome-Scale Analysis of the WRI-Like Family in Gossypium and Functional Characterization of GhWRI1a Controlling Triacylglycerol Content
Source: Front Plant Sci. 2018 Oct 16;9:1516. doi: 10.3389/fpls.2018.01516 (PMC6198791; doi:10.3389/fpls.2018.01516)
Supplement: TABLE S1 — Primers used in this paper. [file Table_1.DOCX]

**Additional file 1: Table S1.** Primers used in this paper.

| **Number** | **Name** | **Sequence (5′-3′)** |
| --- | --- | --- |
| 1 | *GhWRI1a*-L | ATGAAGAGGTCACCGAGTTGTTC |
| 2 | *GhWRI1a*-R | TTAAACAGAGTAGTTACAAGAAAC |
| 3 | Q-*GhWRI1a*-L | GGAAGACGATAATAGGAAGGAGAG |
| 4 | Q-*GhWRI1a*-R | CAGAGTAGTTACAAGAAACCGAGG |
| 5 | Infusion-*GhWRI1a*-L | GGACTCTAGAGGATCCATGAAGAGGTCACCGAGTTGTTC |
| 6 | Infusion-*GhWRI1a*-R | GATCGGGGAAATTCGAGCTCTTAAACAGAGTAGTTACAAGAAAC |
| 7  8 | CRISPR/Cas9-L  CRISPR/Cas9-R | ACTACTTCCTCTCCTATTCAGTCGG  TCTTCCGTTGTGGTGATGC |
| 9 | *GhHis3*-L | GAAGCCTCATCGATACCGTC |
| 10 | *GhHis3*-R | CTACCACTACCATCATGGC |
| 11 | *18S rRNA*-L | *ACATCCAAGGAAGGCAGCA* |
| 12 | *18S rRNA*-R | *TAAGACCAGGAGCGTATCGC* |
