# Supplementary material for: Genome-Scale Analysis of the WRI-Like Family in Gossypium and Functional Characterization of GhWRI1a Controlling Triacylglycerol Content
Source: Front Plant Sci. 2018 Oct 16;9:1516. doi: 10.3389/fpls.2018.01516 (PMC6198791; doi:10.3389/fpls.2018.01516)
Supplement: FILE S1 — Phylogenetic data of Figure 1. [file Data_Sheet_1.docx]

**Additional file S1.** Phylogenetic data of Figure 1.

>AtWRI1/AT3g54320

MKKRLTTSTCSSSPSSSVSSSTTTSSPIQSEAPRPKRAKRAKKSSPSGDKSHNPTSPASTRRSSIYRGVTRHRWTGRFEAHLWDKSSWNSIQNKKGKQVYLGAYDSEEAAAHTYDLAALKYWGPDTILNFPAETYTKELEEMQRVTKEEYLASLRRQSSGFSRGVSKYRGVARHHHNGRWEARIGRVFGNKYLYLGTYNTQEEAAAAYDMAAIEYRGANAVTNFDISNYIDRLKKKGVFPFPVNQANHQEGILVEAKQEVETREAKEEPREEVKQQYVEEPPQEEEEKEEEKAEQQEAEIVGYSEEAAVVNCCIDSSTIMEMDRCGDNNELAWNFCMMDTGFSPFLTDQNLANENPIEYPELFNELAFEDNIDFMFDDGKHECLNLENLDCCVVGRESPPSSSSPLSCLSTDSASSTTTTTTSVSCNYLFQGLFVGSE

>AtWRI2/AT2g41710

MASVSSSDQGPKTEAGCSGGGGGESSETVAASDQMLLYRGFKKAKKERGCTAKERISKMPPCTAGKRSSIYRGVTRFGCELGVVLSVLEYGMFGLVDMHFVSRLKLEEMSRHRWTGRYEAHLWDKSTWNQNQNKKGKQVYLGAYDDEEAAARAYDLAALKYWGPGTLINFPVTDYTRDLEEMQNLSREEYLASLRRKSSGFSRGIAKYRGLQSRWDASASRMPGPEYFSNIHYGAGDDRGTEGDFLGSFCLERKIDLTGYIKWWGANKNRQPESSSKASEDANVEDAGTELKTLEHTSHATEPYKAPNLGVLCGTQRKEKEISSPSSSSALSILSQSPAFKSLEEKVLKIQESCNNENDENANRNIINMEKNNGKAIEKPVVSHGVALGGAAALSLQKSMYPLTSLLTAPLLTNYNTLDPLADPILWTPFLPSGSSLTSEVTKTETSCSTYSYLPQEK

>AtWRI3/AT1g16060

MFIAVEVSPVMEDITRQSKKTSVENETGDDQSATSVVLKAKRKRRSQPRDAPPQRSSVHRGVTRHRWTGRYEAHLWDKNSWNETQTKKGRQVYLGAYDEEDAAARAYDLAALKYWGRDTILNFPLCNYEEDIKEMESQSKEEYIGSLRRKSSGFSRGVSKYRGVAKHHHNGRWEARIGRVFGNKYLYLGTYATQEEAAIAYDIAAIEYRGLNAVTNFDISRYLKLPVPENPIDTANNLLESPHSDLSPFIKPNHESDLSQSQSSSEDNDDRKTKLLKSSPLVAEEVIGPSTPPEIAPPRRSFPEDIQTYFGCQNSGKLTAEEDDVIFGDLDSFLTPDFYSELNDC

>AtWRI4/AT1g79700

MAKVSGRSKKTIVDDEISDKTASASESASIALTSKRKRKSPPRNAPLQRSSPYRGVTRHRWTGRYEAHLWDKNSWNDTQTKKGRQVYLGAYDEEEAAARAYDLAALKYWGRDTLLNFPLPSYDEDVKEMEGQSKEEYIGSLRRKSSGFSRGVSKYRGVARHHHNGRWEARIGRVFGNKYLYLGTYATQEEAAIAYDIAAIEYRGLNAVTNFDVSRYLNPNAAADKADSDSKPIRSPSREPESSDDNKSPKSEEVIEPSTSPEVIPTRRSFPDDIQTYFGCQDSGKLATEEDVIFDCFNSYINPGFYNEFDYGP

>Gh_D10G2551

MKRSPSCSSSSNSCFALPSPSSSSLSASPSSSSSSSSCENPHDQSEKPKAKRARKHQNTDNNACLNNANNNGGRRSSIYRGVTRHRWTGRFEAHLWDKSSWNNIQNKKGRQGAYDSEEAAARTYDLAALKYWGAETILNFPKERYEKEMEEMKKVTKEEYLASLRRRSSGFSRGVSKYRGVARHHHNGRWEARIGRVFGNKYLYLGTYNTQEEAAAAYDMAALEYRGANAVTNFDISHYIERLKQKGILLVDRTEEQIPNPDEARRVESEENGPQPLQEQQERQEKQEQELNQEEAEKSQHFQYMQMQLPLCIDSPMTTMAGIEPTDSNELAWSFCMDSGLTSFLVPDIPLDGTAELPNLFDHDTGFEDNFDLIFDVGPPNKEEANRKCVMDDDVIGVGVSMSMEDNNRKERLSSPSSDSPCSSSTTSVSCNYSV

>Gh_A10G1731

MKRSPSCSSSSNSCFALPSPSSSSSSPSPSSSSSSSSCENPHDLSEKPKAKRGRKHQNTDNNACLNNANNNSGRRSSIYRGVTRHRWTGRFEAHLWDKSSWNNIQNKKGRQGAYDSEEAAARTYDLAALKYWGAETILNFPKERYEKEMEEMKKVTKEEYLATLRRRSSGFSRGVSKYRGVARHHHNGRWEARIGRVFGNKYLYLGTYNTQEEAAAAYDMAALEYRGANAVTNFDISHYIERLKQKGILLVDRTEEQIPNPDEARRVESKENGPQPLQEQQEQQEKQEQELNQEEAEKSQHFQYMQMQLPLCIDSPMTTMAGIEPTDSNELAWSFCMDSGLTSFLVPDIPLDGTAELPNLFDHDAGFEDNFDLIFDVGPPNKEEANRKCMMDEDVIGVGVSMNVEDDNRKERLSSLSSDSPCSSTTSVSCNYSV

>Gh_A05G0024

MTKLSQVNQKNSAQSDSVSNNISTSNDVTKVKKRTRRSFPRDSPPQRSSIYRGSCMLIIWLVNFRHRWTGRFEAHLWDKNCWNESQNKKGRQGAYDDEESAAHAYDLAALKYWGQDTILNFPVSTYQKELKEMENQSREEYIGSLRRKSSGFSRGVSKYRGVARHHHNGRWEARIGRVFGNKYLYLGTYATQEEAATAYDMAAIEYRGLNAVTNFDLSRYIKWLKPNQTNPTTPNSNIDTTTASKLANPSHHQQHNPCFFTTTDDDNQQPQGTGVGIPSETLLTQPRPGNATSALGLLLQSSKFKEMMEMTSAVVVDQCQFTPPPSTTSEPTRYSFPENVETYLECQDSSSLADEDRDIIFGELNSFMAPMFSCDIDA

>Gh_D05G0071

MTKLSQVNQKNSAQSGSVNNNISTSNDVTKVKKRTRRSFPRDSPPQRSSIYRGSCMLIIWIVNFRHRWTGRYEAHLWDKNCWNESQNKKGRQGAYDDEESAAHAYDLAALKYWGQDTILNFPVSTYQKELKEMENQSREEYIGSLRRKSSGFSRGVSKYRGVARHHHNGRWEARIGRVFGNKYLYLGTYATQEEAATAYDMAAIEYRGLNAVTNFDLSRYIKWLKPNQTNPTTPNSNIDATTASTLASPSHHQQHNPSFFTTTDDDNRQPQGTCVGIPSETLLTQPRPGNATSALGLLLQSSKFKEMMEMTSAVVVDQCQLTPPPSTTSEPTRYSFPENVETYLECQDSSSLADEDRDIIFGELNSFMAPMFSCDIDA

>Gh_D12G1652

MEIVTAKSEFRPGRTRLCTAEDNAIDTKCIKRRRRDHSNGALGLSNQQQQHQQLQGDQPTATTVKRSSRFRGVSRHRWTGRFEAHLWDKGSWNPTQRKKGKQGAYDEEESAARAYDLAAIKYWGTSTFTNFPVSDYGTEIEIMRSVTKEEYLASLRRRSSGFSRGVSRYRGVARHHHNGRWEARIGRVFGNKYLYLGTYSTQEEAAHAYDIAAIEYRGINAVTNFDLSTYIRWLKPGANDALISEQIKTASATRPMMTSNIFPTEQTNGLTLFNSNPLTEEAIDIRKKGVVSPCPKSSPALSLLLRSSMFNKLVEQNLNANYDQTEEKMAVGKNGGGEMLCNEVDGGVLPFMCSNNTGLESKESKVPLYNKTGQSMWNGALNLLTNA

>Gh_A12G1529

MEIVTAKSEFSPGRTRLCTAEDNAIDTNYIKRRRRDHSNSALGLSNQQQRHQQLQGDQPTATTVKRSSRFRGVSRHRWTGRFEAHLWDKGSWNPTQRKKGKQGAYDEEESAARAYDLAAIKYWGTSTFTNFPVSDYGTEIEIMRSVTKEEYLASLRRRSSGFSRGVSRYRGVARHHHNGRWEARIGRVFGNKYLYLGTYSTQEEAAHAYDIAAIEYRGINAVTNFDLSTYIRWLKPGANDALISEQIKTASTTRLMMTSNIFPTEQTNGLTLFNSNPLTEKAIDIRKKGVVSPCPKTSPALSLLLRSSMFNKLVEQNLNANYDQTEEKDVKEAVDKNGRGEMLCNEVDGGVLPFMCSNNRGLESKESKVPLYNRTGQSMWNGALNLLTNA

>Gh_A05G0999

MAKISHQNQKNGSDNEKTAAQPTTKLKRTRKTVPRHSPSQRSSTYRGVTRHRWTGRFEAHLWDKNCWNESQNKKGRQGAYADEEAAAHAYDLAALKYWGQDTVLNFPLSTYEKELKEMESQSKEEYIGSLRRKSSGFARGVSKYRGVARHHHNGRWEARIGRVFGNKYLYLGTYATQEEAAMAYDMAAIEYRGLNAVTNFDLSRYIDWLHPNDQSDSNNSSNPQQNFNGDTNSTPSPNHDTKLEISIQSQTYCTSETRLNDSNSNGSSSSASSALGHLLKSSKIKEMLDRTSEAACPSTPPEPNVPRRSFPDYIQTYFDCQDSSSYTEDDDIIFGDLDSLAIPMFHCELDG

>Gh_D05G1117

MAKISHQDQKNGSGNEKTAAQPTTKLKRTRKTVPRHSPPQRSSTYRGVTRHRWTGRFEAHLWDKNCWNESQKKKGRQGAYADEEAAAHAYDLAALKYWGQDTVLNFPLSTYEKELKEMESQSKEEYIGSLRRKSSGFARGVSKYRGVARHHHNGRWEARIGRVFGNKYLYLGTYATQEEAAMAYDMAAIEYRGLNAVTNFDLSRYIGWLHPNDQSDSNNSSNPQQNFNGDTNSTPSPNHDTKLEISIQSQTYCTSDTRLDDSNSNGSSSSASSALGHLLKSSKIKEMLDRTSEAACPSTPPEPNVPRRSFPDYIQTYFDCQDSSSYTEDDDIIFGDLDSLAMPMFHCELDG

>Gh_D09G0206

MEMIMVKDESYQRRRRMSSVYGDVQAVKCVKRRRRDRCDVNQGLQQNDQSSNAPAAAITVKRSSRFRGVSKHRWTGRYEAHLWDKLSWNVTQKKKGKQGAYDDEEAAARAYDLAALKYWGTSTSTNFPISDYEKEIEVMQTVTKEEFLASLRRKSSGFSRGVSKYRGVARHHHNGRWEARIGRVFGNKYLYLGTYNTQEEAARAYDIAAIEYRGINAVTNFDLSTYVGWLRPGMTNNYRIAANETPETVEPESVQSTSCYSPIEESKPSIHYPFATDYFNSPQKQQHVVETKLPVSYKSSSPTALSLLLRSSVFRELVEKNANNVSEDESSNSDADDEQKNQQSGRSDHSDEFGRLFYDEIGSGFSLFFSPTKDSIQLQENELPFVI

>Gh_A09G0218

MEMIMVKDESYQRRRRMSSVYGDVQAVRCVKRRRRDRCDVNQGLQQNDQSRNAPAAAITVKRSSRFRGVSKHRWTGRYEAHLWDKLSWNVTQKKKGKQGAYDDEEAAARAYDLAALKYWGTSTSTNFPISDYEKEIEVMQTVSKEEFLASLRRKSSGFSRGVSKYRGVARHHHNGRWEARIGRVFGNKYLYLGTYNTQEEAARAYDIAAIEYRGINAVTNFDLSTYVGWLKPRMTNNYRIAANETPATVEPESVQSTSCYSPIEESKPSIHYPFAADYFNSPQKQQHVVETNLPVSYKSSSPTALSLLFRSSVFRELVEKNANNVSEDESSNSDADDEQKNQQPGRSDHSDEFGRLFYDEICSGFPLFFSPTKDSIQLQENELPFVI

>Gh_D04G0842

MAKLSQQNHKNTTQNNTSTASNGVTKVKRTRRSVPRDSPPQRSSIYRGYCFRHRWTGRYEAHLWDKNCWNESQNKKGRQGAYDDEEAAAHAYDLAALKYWGQDTILNFPLSTYQKELKEMEDQSREEYIGSLRRKSNGFSRGVSKYRGVARHHHNGRWEARIGRVFGNKYLYLGTYATQEEAATAYDMAAIEYRGLNAVTNFDLSRYIKWLKPNQTKPENNPNPNPNIIDTTTTSLVTPNPDQELDLTFFNGNNHNQQLQESDVISETLLTQPRPVNATSALGLLLQSSKFKEMLEMTSAATDHRQSTPMVSEPVRCGFPEDIQTYFECQDSSCYGNGDDLIFGELNSFGPSMFQCDQLDA

>Gh_A07G1973

MEMMMVNKAQCLGSHLRRLCSVVDDEVQAVRCVKRRRRTPGSVAVGFDGNQGLVQVRPQQQNDQRPIAATTVKRSSRFRGVSRHRWTGRYEAHLWDKLSWNVTQKKKGKQGAYDDEEAAARAYDLAALKYWGTSTFTNFSISDYEKEIEIMQTVTKEEYLASLRRRSSGFSRGVSKYRGVARHHHNGRWEARIGRVFGNKYLYLGTYSTQEEAARAYDIAAIEYRGINAVTNFDLSTYFRWLKPGTMVEPESKPSFHHSLPTDYLKSPEKQEVFKTKTPSSSSSSPTALDLLFRSSIFRELVKKNSNVSSEDGSSVTDGDDESKNQQGGNGVDADDEFSRLFYDGIGDFPFMCSSTKSSIELKHM

>Gh_A05G3160

MDMEMGMVNDEQCLGLGDSQNVQVEGIRCAKRRRRDPAVSVAFDNRDGHQQRNAAATATATTVKRSSRFRGVSRHRWTGRYEAHLWDKLSWNISQKKKGKQGAYDEEEAAARAYDLAALKYWGTSTFTNFPISDYAKEIEIMQTLTKEEYLASLRRKSSGFSRGVSKYRGVARHHNNGRWEARIGRVFGNKYLYLGTYSTQEEAARAYDIAAIEYRGINAVTNFDLSTYIRWLKPNESLPMAVEPEPVILPSQASTPGEESKPSVNHSSTADYLNSSPKQVVESKIHVMNSNKCSSTTALGLLLRSSIFRDLVEKNVANVCEDESGSPDENEEKNKHLAGNDDEFCGLFYNGIGTEFPFFRSSMKDTMELQERGSSFI

>Gh_A04G1351

MAKLSQQNHKNTTQNNTSTASNGVTKVKRTRRSVPRDSPPQRSSIYRGVTRQYCFRHRWTGRYEAHLWDKNCWNESQNKKGRQGAYDDEAAAAHAYDLAALKYWGQDTILNFPVSLSLIFIYMVCGRIEEETYLTSTMQLSTYQKELKEMEDQSREEYIGSLRRKSSGFSRGVSKYRGVARHHHNGRWEARIGRVFGNKYLYLGTYATQEEAATAYDMAAIEYRGLNAVTNFDLSRYIKWLKPNQTKPENNPNPNPNIIDTTTTSLVTTNPDQELDLTFFNGNNHNQKLQESDVISETLLTQPRPVNATSALGLLLQSSKFKEMMEMTSAATDHRQSTSMISEPVRCGFPEDIQTYFECQDSSCYGNGDDLIFGELNSFGPSMFQCDQLDA

>Gh_A09G0219

MVKDEQNDDNQGMQQNDQSSNVATTVKRSSRFRGVSRHRWTGKYEAHLWDKLSWNVTQKKKGKQGAYDDEEVAARAYDLAALKYWGTPTSTNFPISYYEKEIEIMQTVTKEEFLASLRRKSNGFSRGVSKHYYHNGRSEARIGRVFGNKYLNLGSYNTQEEAARVYDIAAIKCRGINTVTNFDLSTYAGWLRPELTNNTETESVQSTSCYSRLEQSKPSIHYPFAVDHFNSLKQEHVETKLPVINVKSYKSSSPTALSLLLRSSVFQELVEKNANNRK

>Gh_D04G0466

MEMRMVNDERYLGLGRRRKCCDDSENIQVEAIRCAKRRRRDPAGSVAFDNRDGHQQRNAAATATTVKRSSRFRGVSRYCKIKQVFGDIQNYNNHRINMKMNRHRWTGRYEAHLWDKLSWNITQKKKGKQGAYDEEEAAARAYDLAALKYWGTSTFTNFPISDYGKEIEIMQTLTKEEYLASLRRKSSGFSRGVSKYRGVARHHNNGRWEARIGRVFGNKYLYLGTYSKFHIWTPGRYIFVSNTYTGLYISFTGTQEEAARAYDIAAIEYRGINAVTNFDLSTYIRWLKPNESLPMAVEPEPVTLPSQASTPREESKPSFNHSSTADYLNSFPKQVVESKIHVMNSNKCSSTTALGLLLRSSIFRDLVEKNLANVCEDESGSTDEDEEKNKHLAGNDDEFCGLFYDGIGTGFPFFRSSMKDTMELQERGSSFI

>Gh_D07G2191

MEMMMVNKAQCLGSHRRRLCSVVDDEVQAVRCVKRRRRTPDSVAVGFDGNQGLVQVQPQQQNEQRPIAATTVKRSSRFRGVSRHRWTGRYEAHLWDKLSWNVTQKKKGKQGFGVLSFWSIDAYVLLYDFMFFHWFLLWISLLGILQFILISDYEKEIEIMQTVTKEEYLASLRRRSSGFSRGVSKYRGVARHHHNGRWEARIGRVFGNKYLYLGTYSTQEEAARAYDIAAIEYRGINAVTNFDLSTYFRWLKPGTMVEPESKPSFHHSLPTDYLKSPEKQEVFKTKTPSSSSSSPTALDLLFRSSIFRELVKKNSNVSSEDGSSVTDGDDESKNQQGGNGVDADDEFSRLFYDGIDDFPFMCSSTKSSIELKHI

>Gh_A13G0020

MNTSKNNSKSKFKQKQNKVELLLLILYVIGFYIFCIHRSLRLSRDHFSKLRGLRVGWLFSPARLNDASDDQWRNFRTNLPILSLVFGIFTLLANIFRKLFRLRARGMSFLWFFLSLIYLSYLHGACILFIFLIASSNFLLVKIFARAKYFTFLLWIFNLGFLFCNRIYQGYSFSIFGEYWEYLDNFRGTFRWHICFNLVVLRMISFGYDYHWAHQESRFDQEKHIQRCHVCKSGKNCYQILQERNAHMNDYTFTTYLSYLVYAPLYIAGPIISFNSFASQLDVPQNHYSIKEVIWYGLRWVFGLCLMELMTHLFYFNAFATSASWKMLSPMDIFIVGYGVINFMWLKFFLIWRFFRFCSLIAGIEAPENMPKCINNCHDLESFWKSWHASFNKWIVRYMYIPLGGSQRKLINIWVIFTFVAVWHDLEWKLLSWAWLTCLFFVPELLVKSATNAFQAKGALDGFIFRELRAAGGTITITCLMVANLVGYVIGPSGFSWLISQFLSKEGAYDSEEDAARTYDLAALKYWGPETTLNFSVERYGKEIEEMNKVSKEEYLASLRRRSSGFSRGISKYRGVASRHHHNGRWEARIGRVFGNKYLYLGTYNTEEEAAAAYDMAALEYRGVNAITNFDISHYVERLKEKGILFLDPTPEQSPRSVEVGPIEVEQQPQQGYEAADEHQHFQNMQMQLPLCNDNATTMVGTETTDGNELAWSFCMDSGLTSFFSPEFPNVFDDIGFEDNIDSLFNLGNNKNAVGRKCLSDEASCVEVGDSSTTFL

>Gh_D03G0620

MASTSSSDPGMKAEASGGENSETVIANDQLLLCRGLKKAKKERGCTAKERISKMPPCTAGKRSSIYRGVTRHRWTGRYEAHLWDKSTWNQNQNKKGKQGLSQHIFSGAYDDEEAAARAYDLAALKYWGPGTLINFPVTDYTRDLEEMQNVSREDYLASLRRKSSGFSRGISKYRGLSSRRWDSSFGRVSGSEYFNSIHYGDDTTKENDYIGGFCIERKIDLTGYIKWWGTNKTRQAEAGTKSSEETKNACPEDIGSELKTSEWAVQPTEPYQMPRLGTSLEGTKCKGSSVSALSILSRSADFKSLQEKALKKQEQNSDNDENENKNTINKMDYGKAAEKSVNNDTGGDRLGAAMGMTGGLSSLQKNVFPLTPFLSAPLLTNYNTIDSLVDPVLWTSLVPALPTGPSRNPEVTKTETSSTYTFFRPEE

>Gh_A02G1061

MASTSSSDPGMKAEASGGENSETVIANDQLLLCRGLKKAKKERGCTAKERISKMPPCTAGKRSSIYRGVTRHRWTGRYEAHLWDKSTWNQNQNKKGKQGLSACLSFQHIFSGAYDDEEAAARAYDLAALKYWGPGTLINFPVTDYTRDLEEMQNVSREDYLASLRRKSSGFSRGISKYRGLSSRRWDSSFGRVSGSEYFNSIHYGDDTTKENDYIGGFCIERKIDLTGYIKWWGTNKTRQAEAGTKSSEETKNACPEDIGSELKTSEWAVQPTEPYQMPRLGTSLEGTKCKGSSVSALSILSRSADFKSLQEKALKKQEQNSDSDENENKNTINKMDHGKAAEKSVNHDTGGDRLGAAMGMTGGLSSLQKNVYPLTPFLSVPLLTNYNTIDSLVDPVLWTSLVPALPTGPSRNPEVTKTETSSTYTFFRPEE

>Gh_D13G0036

MEEEVNLSFKEAKLAWDRRSPQNSLEYTNLFVGILLTLASTYELVRPSPETTLPTLKIETSPGNASKTSSKELSQHRGGVAGKSVPPNSFDKSKVLDVKPLRSLLPVFPEAPNGPPFVCAPPNGPFPTGFSPFFPFSGPQGSPLTPGLNQNLFNSTAMPIRSFRAEPPPASNGENVQSSNKRKSVGPSFVKKKVKRSNDSELALAALTNFKPGISAAEKDDGNRELVENVLMRFEALRRKLSQMEDAKESHSDIFKRANLKAGNIMFTKGVRTNGKKRIGAVPGVEIGDIFFFRMELILIGLHSQSMAGIDFMPMKADIEGERVAISIVSSGGYEDNAEDPDVLVYTGQGGNASADKEASDQKLVRGNIALERSLHRANEVRVIRGFKDATHQTSKVYVYDGLYKVQESWMEKGKTGCNMFKYKLVRLPGQTGAFSTWKSIRKWKEDPSSRDGLILPDLTSGAESIPVSLVNEVDDEKGPAYFTYVSTVKYPKSFKLVQPSYGCNCRDACQAGNSNCSCIQKNGGDFPYITTGILACRMPMIFECGSSCPCFRNCKNRVLQTGFKVHFEVFKTRDKGWGLRSWDPIRAGTFICEYAGEVIEKIKEKADGDDGENNDYVFDTNRVYESFKWNHETESAEERSDTSEKFDIPSPLIISSKNSGNIARFMNHSCSPNVFWQPIMYEHNNEAFLHIAFFAKKHIPPMTELTYDYGIPRSDETESNNMEHGKKKCLCGSPKCRDHFSKLRGLRIGWLFSPPRLNDASDDQWRNFRTNLPILSLVFGIFTLLANIFRKLFHLRARGMSFFWLFLSLIYISYLHGACILFIFLIASSNFLLVKIFARAKYFTFLLWIFNLGFLFCNRIYQGYSFSIFGEYWEYLDNFRGTFRWHICFNLVVLRMISFGYDYHWAHQESRFDQEKHIQRCHVCKSGKNCYQILQERNAHINDYSFTTYLSYLVFAPLYIAGPIISFNSFASQLDVPQNHYSIKEVIWYGLRWVFGLCLMELMTHLFYFNAFATSASWKMLSPMDIFIIGYGVINFMWLKFFLIWRFFRFCSLIAGIEAPENMPKCVNNCHDLESFWKSWHASFNKWIVRYMYIPLGGSQRKLLNIWVIFTFVAVWHDLEWKLLSWAWLTCLFFVPELLVKSATNAFQAKGALDGFIFRELRAAGGTITITCLMVANLVGYVIGPSGFSWLISQFLSKEGLNVFGFMLLTFYVGTKGNRLESYVFPFLSNPTHISEQQGSKKVLRMTVSCDATRIMMALESPSTSIHRKLNSQANFSIYKHVMTSIRLLYLNVVISSYLMNGQTSYAWKYTHVIHYLYVASLSSPSSPSSSSLSSSSSSESPQNVVSVISEKPRGKRVERYGKEIEEMNKVSKEEYLASLRRRSSGFSRGISKYRGVASRHHHNGRWEARIGRVFGNKYLYLGTYNTEEEAAAAYDMAALEYRGVNAVTNFDISHYVERLKEKGILYLDPTPEQSPSSVEVGPIEVEQQPQQGYEAADEHQHFQNMQMQLPLCNDNATTMVGTETTDGNELAWSFCMDSGLTSFFSPEFPNVFDDMGFEDNVDSLFDLGNNKNAVGRKCLSDEASCVEVGDSSTTSVSCDGFSFWG

>Gh_D09G0207

MQTVTKEEFLASLRRKSSGFSRGVSKYRGVAKHYYHNGRWEARIGREFGNKYLNLGSYNTQEEAARAYDIAAIKCRGINAVTNFDLSTYAGWLRPELTNNTETESVQSTSCYSLIEQSKPSIHYPFAVGHFNSLKQEHAETKLPVINVKSYKSSSPTALSLLLRSSVFQELVEKNANNRK

>Gbscaffold14438.14.2

MKRSPSCCSSSNSCFALPSPSSSSLSPSPSSSSSSSSCENPHDQSEKPKAKRARKHQNTDNNACLNNANNNGGRRSSIYRGVTRHRWTGRFEAHLWDKSSWNNIQNKKGRQVYLGAYDSEEAAARTYDLAALKYWGAETILNFPKERYEKEMEEMKKVTKEEYLASLRRRSSGFSRGVSKYRGVARHHHNGRWEARIGRVFGNKYLYLGTYNTQEEAAAAYDMAALEYRGANAVTNFDISHYIERLKQKGILLVDRTEEQIPNPDEARRVESKENGPQPLQEQQEQQEKQEQELNQEEANRKCVMDDDVIGVGVSMSMEDNNRKERLSSPSSDSPCSSSTTSVSCNYSV

>Gbscaffold14438.14.0

MKRSPSCCSSSNSCFALPSPSSSSLSPSPSSSSSSSSCENPHDQSEKPKAKRARKHQNTDNNACLNNANNNGGRRSSIYRGVTRHRWTGRFEAHLWDKSSWNNIQNKKGRQVYLGAYDSEEAAARTYDLAALKYWGAETILNFPKERYEKEMEEMKKVTKEEYLASLRRRSSGFSRGVSKYRGVARHHHNGRWEARIGRVFGNKYLYLGTYNTQEEAAAAYDMAALEYRGANAVTNFDISHYIERLKQKGILLVDRTEEQIPNPDEARRVESKENGPQPLQEQQEQQEKQEQELNQEEAEKSQHFQYMQMQLPLCIDSPMTTMAGIEPTDSNELAWSFCMDSGLTSFLVPDIPLDGTAELPNLFDHDTGFEDNFDLIFDVGPPNKEEANRKCVMDDDVIGVGVSMSMEDNNRKERLSSPSSDSPCSSSTTSVSCNYSV

>Gbscaffold22373.8.1

MKRSPSCSSSSNSCFALPSPSSSSSSPSPSSSSSSSSCENPHDLSEKPKAKRARKHQNTDNNACLNNANNNSGRRSSIYRGVTRHRWTGRFEAHLWDKSSWNNIQNKKGRQVYLGAYDSEEAAARTYDLAALKYWGAETILNFPKERYEKEMEEMKKVTKEEYLATLRRRSSGFSRGVSKYRGVARHHHNGRWEARIGRVFGNKYLYLGTYNTQEEAAAAYDMAALEYRGANAVTNFDISHYIERLKQKGILLVDRTEEQIPNPDEARRVESKENGPQPLQEQQEQQEKQEQELNQEEAEKSQHFQYMQMQLPLCIDSPMTTMAGIEPTDSNELAWSFCMDSGLTSFLVPDIPLDGTAELPNLFDHDAGFEDNFDLIFDVGPPNKEEANRKCMMDEDVIGVGVSMNVEDDNRKERLSSLSSDSPCSSTTSVSCNYSV

>Gbscaffold20501.18.0

MKRSSTCSSSSNSSIASPSSPSSPSSSSLSSSSSSSESPQNVVSVISEKPKGKRVRKNQNQKCISSNANTSRRSSIYRGVTRHRWTGRFEAHLWDKTSWNSIQNKKGRQVYLGAYDSEEDAARTYDLAALKYWGPETTLNFPVERYGKEIEEMNKVSKEEYLASLRRRSSGFSRGISKYRGVARHHHNGRWEARIGRVFGNKYLYLGTYNTEEEAAAAYDMAALEYRGVNAVTNFDISHYVERLKEKGILYLDPTPEQSPSSVEVGPIEVEQQPQQGYEAADEHQHFQNLQMQLPLCNDNATTMVGTETTDGNELAWSFCMDSGLTSFFSPEFPNVFDDMGFEDNVDSLFDLGNNKNAVGRKCLSDEASCVEVGDSSTTSVSCDGFSFWG

>Gbscaffold1205.6.1

MAKLSQQNHKNTTQNNTSTASNGVTKVKRTRRSVPRDSPPQRSSIYRGVTRHRWTGRYEAHLWDKNCWNESQNKKGRQVYLGAYDDEEAAAHAYDLAALKYWGQDTILNFPLSTYQKELKEMEDQSREEYIGSLRRKSSGFSRGVSKYRGVARHHHNGRWEARIGRVFGNKYLYLGTYATQEEAATAYDMAAIEYRGLNAVTNFDLSRYIKWLKPNQTKPENNPNPNPNIIDTTTTSLVTPNPDQELDLTFFNGNNHNQQLQESDVISETLLTQPRPVNATSALGLLLQSSKFKEMMEMTSAATDHRQSTPMVSEPVRCGFPEDIQTYFECQDSSCYGNGDDLIFGELNSFGPSMFQCDQLDA

>Gbscaffold25274.2.0

MAKLSQQNHKNTTQNNTSSASNGVTKVKRTRRSVPRDSPPQRSSIYRGVTRHRWTGRYEAHLWDKNCWNESQNKKGRQVYLGAYDDEAAAAHAYDLAALKYWGQDTILNFPLSTYQKELKEMEDQSREEYIGSLRRKSSGFSRGVSKYRGVARHHHNGRWEARIGRVFGNKYLYLGTYATQEEAATAYDMAAIEYRGLNAVTNFDLSRYIKWLKPNQTKPENNPNPNPNIIDTTTTSLVTPNPDQELDLTFFNGNNHNQKLQESDVISETC

>Gbscaffold2524.5.0

MAKISHQNQKNGSDNEKTAAQPTTKLKRTRKTVPRHSPSQRSSTYRGVTRHRWTGRFEAHLWDKNCWNESQNKKGRQVYLGAYADEEAAAHAYDLAALKYWGQDTVLNFPLSTYEKELKEMESQSKEEYIGSLRRKSSGFARGVSKYRGVARHHHNGRWEARIGRVFGNKYLYLGTYATQEEAAMAYDMAAIEYRGLNAVTNFDLSRYIDWLHPNDQSDSNNSSNPQQNFNGDTNSTPSPNHDTKLEISIQSQTYCTSETRLNDSNSNGSSSSASSALGHLLKSSKIKEMLDRTSEAACPSTPPEPNVPRRSFPDYIQTYFDCQDSSSYTEDDDIIFGDLDSLAIPMFHCELDG

>Gbscaffold12660.29.0

MAKISHQDQKNGSGNEKTAAQPTTKLKRTRKTVPRHSPPQRSSTYRGVTRHRWTGRFEAHLWDKNCWNESQKKKGRQVYLGAYADEEAAAHAYDLAALKYWGQDTVLNFPLSTYEKELKEMESQSKEEYIGSLRRKSSGFARGVSKYRGVARHHHNGRWEARIGRVFGNKYLYLGTYATQEEAAMAYDMAAIEYRGLNAVTNFDLSRYIGWLHPNDQSDSNNSSNPQQNFNGDTNSTPSPNHDTKLEISIQSQTYCTSDTRLDDSNSNGSSSSASSALGHLLKSSKIKEMLDRTSEAACPSTPPEPNVPRRSFPDYIQTYFDCQDSSSYTEDDDIIFGDLDSLAMPMFHCELDG

>Gbscaffold259.12.0

MEIVTAKSEFRPGRTRLCTAEDNAIDTKCIKRRRRDHSNGALGLSNQQQQHQQLQGDQPTATTVKRSSRFRGVSRHRWTGRFEAHLWDKGSWNPTQRKKGKQGAYDEEESAARAYDLAAIKYWGTSTFTNFPVSDYGTEIEIMRSVTKEEYLASLRRRSSGFSRGVSRYRGVARHHHNGRWEARIGRVFGNKYLYLGTYSTQEEAAHAYDIAAIEYRGINAVTNFDLSTYIRWLKPGANDALISEQIKTASATRPMMTSNIFPTEQTNGLTLFNSNPLTEEAIDIRKKGVVSPCPKSSPALSLLLRSSMFNKLVEQNLNANYDQTEEKVAVDKNGGGEMLCNEVDGGVLPFMCSNNTGLESKESKVPLYNKTGQSMWNGALNLLTNA

>Gbscaffold19204.1.0

MEIVTAKSEFSPGRTRLCTAEDNAIDTNYIKRRRRDHSNSALGLSNQQQRHQQLQGDQPTATTVKRSSRFRGVSRHRWTGRFEAHLWDKGSWNPTQRKKGKQGAYDEEESAARAYDLAAIKYWGTSTFTNFPVSDYGTEIEIMRSVTKEEYLASLRRRSSGFSRGVSRYRGVARHHHNGRWEARIGRVFGNKYLYLGTYSTQEEAAHAYDIAAIEYRGINAVTNFDLSTYIRWLKPGANDALILEQIKTASTTRLMMTSNIFPTEQTNGLTLFNSNPLTEKATDIRKKGVVSPCPKTSPALSLLLRSSMFNKLVEQNLNANYDQTEEKDVKEAVDKNGRGEMLCNEVDGGVLPFMCSNNRGLESKESKVPLYNKTGQSMWN

>Gbscaffold12660.29.1

MAKISHQDQKNGSGNEKTAAQPTTKLKRTRKTVPRHSPPQRSSTYRGVTRHRWTGRFEAHLWDKNCWNESQKKKGRQVYLGAYADEEAAAHAYDLAALKYWGQDTVLNFPLSTYEKELKEMESQSKEEYIGSLRSSGFARGVSKYRGVARHHHNGRWEARIGRVFGNKYLYLGTYATQEEAAMAYDMAAIEYRGLNAVTNFDLSRYIGWLHPNDQSDSNNSSNPQQNFNGDTNSTPSPNHDTKLEISIQSQTYCTSDTRLDDSNSNGSSSSASSALGHLLKSSKIKEMLDRTSEAACPSTPPEPNVPRRSFPDYIQTYFDCQDSSSYTEDDDIIFGDLDSLAMPMFHCELDG

>Gbscaffold2524.5.1

MAKISHQNQKNGSDNEKTAAQPTTKLKRTRKTVPRHSPSQRSSTYRGVTRHRWTGRFEAHLWDKNCWNESQNKKGRQGAYADEEAAAHAYDLAALKYWGQDTVLNFPLSTYEKELKEMESQSKEEYIGSLRRKSSGFARGVSKYRGVARHHHNGRWEARIGRVFGNKYLYLGTYATQEEAAMAYDMAAIEYRGLNAVTNFDLSRYIDWLHPNDQSDSNNSSNPQQNFNGDTNSTPSPNHDTKLEISIQSQTYCTSETRLNDSNSNGSSSSASSALGHLLKSSKIKEMLDRTSEAACPSTPPEPNVPRRSFPDYIQTYFDCQDSSSYTEDDDIIFGDLDSLAIPMFHCELDG

>Gbscaffold1804.8.0

MEMMMVNKAQCLGSHLRRLCSVVDDEVQAVRCVKRRRRTPGSVAVGFDGNQGLVQVRPQQQNDQGPIAATTVKRSSRFRGVSRHRWTGRYEAHLWDKLSWNVTQKKKGKQGAYDDEEAAARAYDLAALKYWGTSTFTNFSISDYEKEIEIMQTVTKEEYLASLRRRSSGFSRGVSKYRGVARHHHNGRWEARIGRVFGNKYLYLGTYSTQEEAARAYDIAAIEYRGINAVTNFDLSTYFRWLKPGTMVEPESKPSFHHSLPTDYLKSPEKQEVFKTKTPSSSSSSPTALDLLFRSSIFRELVKKNSNVSSEDGSSVTDGDDESKNQQGGNGVDADDEFSRLFYDGIGDFPFMCSSTKSSIELKHM

>Gbscaffold22373.8.0

MNFILWKTGAYDSEEAAARTYDLAALKYWGAETILNFPKERYEKEMEEMKKVTKEEYLATLRRRSSGFSRGVSKYRGVARHHHNGRWEARIGRVFGNKYLYLGTYNTQEEAAAAYDMAALEYRGANAVTNFDISHYIERLKQKGILLVDRTEEQIPNPDEARRVESKENGPQPLQEQQEQQEKQEQELNQEEAEKSQHFQYMQMQLPLCIDSPMTTMAGIEPTDSNELAWSFCMDSGLTSFLVPDIPLDGTAELPNLFDHDAGFEDNFDLIFDVGPPNKEEANRKCMMDEDVIGVGVSMNVEDDNRKERLSSLSSDSPCSSTTSVSCNYSV

>Gbscaffold17450.12.0

HRWTGRYEAHLWDKLSWNITQKKKGKQGAYDEEEAAARAYDLAALKYWGTSTFTNFPISDYGKEIEIMQTLTKEEYLASLRRKSSGFSRGVSKYRGVARHHNNGRWEARIGRVFGNKYLYLGTY

>Gbscaffold9581.6.0

HRWTGRYEAHLWDKLSWNISQKKKGKQGAYDEEEAAARAYDLAALKYWGTSTFTNFPISDYAKEIEIMQTLTKEEYLASLRRKSSGFSRGVSKYRGVARHLNNGRWEARIGRVFGNKYLYLGTY

>Gbscaffold3103.5.0

MASTSSSDPGMKAEASGGENSETVIANDQLLLCRGLKKAKKERGCTAKERISKMPPCTAGKRSSIYRGVTRHRWTGRYEAHLWDKSTWNQNQNKKGKQVYLGAYDDEEAAARAYDLAALKYWGPGTLINFPVTDYTRDLEEMQNVSREDYLASLRRKSSGFSRGISKYRGLSSRRWDSSFGRVSGSEYFNSIHYGDDTTKENDYIGGFCIERKIDLTGYIKWWGTNKTRQAEAGTKSSEETKNACPEDIGSELKTSEWAVQPTEPYQMPRLGTSLEGTKCKGSSVSALSILSRSADFKSLQEKALKKQEQNSDNDENENKNTINKMDYGKAAEKSVNNDTGGDRLGAAMGMTGGLSSLQKNVFPLTPFLSAPLLTNYNTIDSLVDPVLWTSLVPALPTGPSRNPEVTKTETSSTYTFFRPEE

>Gbscaffold1219.3.0

MASTSSSDPGMKAEASGGENSETVIANDQLLLCRGLKKAKKERGCTAKERISKMPPCTAGKRSSIYRGVTRHRWTGRYEAHLWDKSTWNQNQNKKGKQVYLGAYDDEEAAARAYDLAALKYWGPGTLINFPVTDYTRDLEEMQNVSREDYLASLRRKSSGFSRGISKYRGLSSRRWDSSFGRVSGSEYFNSIHYGDDTTKENDYIGGFCIERKIDLTGYIKWWGTNKTRQAEAGTKSSEETKNACPEDIGSELKTSEWAVQPTEPYQMPRLGTSLEGTKCKGSSVSALSILSRSADFKSLQEKALKKQEQNSDSDENQNKNTINKMDHGKAAEKSVNHDTGGDRLGAAMGMTGGLSSLQKNVYPLTPFLSAPLLTNYNTIDSLVDPVLWTSLVPALPTGPSRNPEVTKTETSSTYTFFRPEE

>Gbscaffold14438.14.1

MEEMKKVTKEEYLASLRRRSSGFSRGVSKYRGVARHHHNGRWEARIGRVFGNKYLYLGTYNTQEEAAAAYDMAALEYRGANAVTNFDISHYIERLKQKGILLVDRTEEQIPNPDEARRVESKENGPQPLQEQQEQQEKQEQELNQEEAEKSQHFQYMQMQLPLCIDSPMTTMAGIEPTDSNELAWSFCMDSGLTSFLVPDIPLDGTAELPNLFDHDTGFEDNFDLIFDVGPPNKEEANRKCVMDDDVIGVGVSMSMEDNNRKERLSSPSSDSPCSSSTTSVSCNYSV

>Gbscaffold18379.5.0

MNNIQVSTYQKELKEMENQSREEYIGSLRRKSSGFSRGVSKYRGVARHHHNGRWEARIGRVFGNKYLYLGTYATQEEAATAYDMAAIEYRGLNAVTNFDLSRYIKWLKPNQTNPTTPNSNIDTTTASKLANPSHHQQHNPGFFTTTDDDNRQPQGTGVGIPSETLLTQPRPGNATSALGLLLQSSKFKEMMEMTSAVVVDQCQFTPPPSTTSEPTRYSFPENVETYLECQDSSSLADEDRDIIFGELNSFMAPMFSCDIDA

>Gbscaffold18152.14.0

MNKVSKEEYLASLRRRSSGFSRGISKYRGVASRHHHNGRWEARIGRVFGNKYLYLGTYNTEEEAAAAYDMAALEYRGVNAITNFDISHYVERLKEKGILFLDPTPEQSPRSVEVGPIEVEQQPQQGYEAADEHQHFQNMQMQLRLCNDNATTMVGTETTDGNELAWSFCMDSGLTSFFSPEFPNVFDDIGFKDNIDSLFDLGNNKNAVGRKCLSDEASCVEVGDSSTTFL

>Gbscaffold18152.14.1

MNKVSKEEYLASLRRRSSGFSRGISKYRGVASRHHHNGRWEARIGRVFGNKYLYLGTYKEEAAAAYDMAALEYRGVNAITNFDISHYVERLKEKGILFLDPTPEQSPRSVEVGPIEVEQQPQQGYEAADEHQHFQNMQMQLRLCNDNATTMVGTETTDGNELAWSFCMDSGLTSFFSPEFPNVFDDIGFKDNIDSLFDLGNNKNAVGRKCLSDEASCVEVGDSSTTFL

>Gbscaffold1205.6.0

MNRHHHNGRWEARIGRVFGNKYLYLGTYATQEEAATAYDMAAIEYRGLNAVTNFDLSRYIKWLKPNQTKPENNPNPNPNIIDTTTTSLVTPNPDQELDLTFFNGNNHNQQLQESDVISETLLTQPRPVNATSALGLLLQSSKFKEMMEMTSAATDHRQSTPMVSEPVRCGFPEDIQTYFECQDSSCYGNGDDLIFGELNSFGPSMFQCDQLDA

>Cotton_A_24703

MKRSPSCSSSSNSCFALPSPSSSSLSPSPSSSSSSSSCENPHDLSEKPKAKRARKHQNTDNNACLNNANNNSGRRSSIYRGVTRHRWTGRFEAHLWDKSSWNNVQNKKGRQVYLGAYDSEEAAARTYDLAALKYWGAETILNFPKERYEKEMEEMKKVTKEEYLATLRRRSSGFSRGVSKYRGVARHHHNGRWEARIGRVFGNKYLYLGTYNTQEEAAAAYDMAALEYRGANAVTNFDISHYIERLKQKGILLVDRTEEQIPNPDEARRVESKENGPQPLQEQQEQREKQEQELNQEEAEKSQHFQYMQMQLPLCIDSPMTTMAGIEPTDSNELAWSFCMDSGLTSFLVPDIPLDGTAELPNLFDHDAGFEDNFDLIFDVGPPNKEEANRKCMMDEDVIGVGVSMNVEDDNRKERLSSLSSDSPCSSTTSVSCNYSV

>Cotton_A_16267

MTKLSQVNQKNSAQSDSVSNNISTSNDVTKVKKRTRRSFPRDSPPQRSSIYRGVTRQHRWTGRFEAHLWDKNCWNESQNKKGRQGAYDDEESAAHAYDLAALKYWGQDTILNFPVSTYQKELKEMENQSREEYIGSLRRKSSGFSRGVSKYRGVARHHHNGRWEARIGRVFGNKYLYLGTYATQEEAATAYDMAAIEYRGLNAVTNFDLSRYIKWLKPNQTNPTTPNSNIDTTTASKLANPSHHQQHNPGFFTTTDDDNRQPQGTGVGIPSETLLTQPRPGNATSALGLLLQSSKFKEMMEMTSAVVVDQCQFTPPPSTTSEPTRYSFPENVETYLECQDSSSLADEDRDIIFGELNSFMAPMFSCDINA

>Cotton_A_06134

MEIVTAKSEFSPGRTRLCTAEDNAIDTNCIKRRRRDHSNSALGLSNQQQRHQQLQGDQPTATTVKRSSRFRGVSRHRWTGRFEAHLWDKGSWNPTQRKKGKQGAYDEEESAARAYDLAAIKYWGTSTFTNFPVSDYGTEIEIMRSVTKEEYLASLRRRSSGFSRGVSRYRGVARHHHNGRWEARIGRVFGNKYLYLGTYSTQEEAAHAYDIAAIEYRGINAVTNFDLSTYIRWLKPGANDALISEQIKTASTTRLMMTSNIFPTEQTNGLTLFNSNPLTEKAIDIRKKGVVSPCPKTSPALSLLLRSSMFNKLVEQNLNANYDQTEEKDVKEAIDKNGRGEMLCNEVDGGVLPFMCSNNRGLESKESKVPLYNKTGQSMWNGALNLLTNA

>Cotton_A_17105

MAKISHQNQKNGSDNEKTAAQPTTKLKRTRKTVPRHSPPQRSSTYRGVTRHRWTGRFEAHLWDKNCWNESQNKKGRQGAYADEEAAAHAYDLAALKYWGQDTVLNFPLSTYEKELKEMESQSKEEYIGSLRRKSSGFARGVSKYRGVARHHHNGRWEARIGRVFGNKYLYLGTYATQEEAAMAYDMAAIEYRGLNAVTNFDLSRYIDWLHPNDQSDSNNSSNPQQNFNGDTNSTPSPNHDTKLEISIQSQTYCTSETRLNDSNSNGSSSSASSALGHLLKSSKIKEMLDRTSEAACPSTPPEPNVPRRSFPDYIQTYFDCQDSSSYTEDDDIIFGDLDSLAIPMFHCELDG

>Cotton_A_28204

MEMIMVKDESYQRRRRMSSVYGDVQAVRCVKRRRRDRCDVNQGLQQNDQSSNAPAAAITVKRSSRFRGVSKHRWTGRYEAHLWDKLSWNVTQKKKGKQGAYDDEEAAARAYDLAALKYWGTSTSTNFPISDYEKEIEVMQTVTKEEFLASLRRKSSGFSRGVSKYRGVARHHHNGRWEARIGRVFGNKYLYLGTYNTQEEAARAYDIAAIEYRGINAVTNFDLSTYVGWLRPGMTNNYRIAANETPATVEPESVQSTSCYSPIEESKPSIHYPFAADYFNSPQKQHVVETNLPVSYKSSSPTALSLLFRSSVFRELVEKNANNVSEDESSNSDADDEQKNQQPGRSDHSDEFGRLFYDEIGSGFPLFFSPTKDSIQLQENELPFVI

>Cotton_A_19437

MMMVNKAQCLGSHLRRLCSVVDDEVQAVRCVKRRRRTPGSVAVGFDGNQGLVQVQPQQQNDQRPIAATTVKRSSRFRGVSRHRWTGRYEAHLWDKLSWNVTQKKKGKQGAYDDEEAAARAYDLAALKYWGTSTFTNFSISDYEKEIEIMQTVTKEEYLASLRRRSSGFSRGVSKYRGVARHHHNGRWEARIGRVFGNKYLYLGTYSTQEEAARAYDIAAIEYRGINAVTNFDLSTYFRWLKPGTMVEPESKPSFHHSLPTDYLKSPEKQEVFKTKTPSSSSSSPTALDLLFRSSIFRELVKKNSNVSSEDGSSVTDGDDESKNQQGGNGVDADDEFSRLFYDGIGDFPFMCSSTKSSIELKHM

>Cotton_A_29003

MDMEMGMVNDEQCLGLGDSQNVQVEGIRCAKRRRRDPAVSVAFDNRDGHQQRNAAATATATTVKRSSRFRGVSRHRWTGRYEAHLWDKLSWNISQKKKGKQGAYDEEEAAARAYDLAALKYWGTSTFTNFPISDYAKEIEIMQTLTKEEYLASLRRKSSGFSRGVSKYRGVARHHNNGRWEARIGRVFGNKYLYLGTYSTQEEAARAYDIAAIEYRGINAVTNFDLSTYIRWLKPNESLPMAVEPEPVILPSQASTPGEESKPSVNHSSTADYLNSSPKQVVESKIHVMNSNKCSSTTALGLLLRSSIFRDLVEKNVANVCEDESGSTDENEEKNKHLAGNDDEFCGLFYNGIGTEFPFFRSSMKDTMELQERGSSFI

>Cotton_A_41232

MAKLSQQNHKNTTQNNTSTASNGVTKVKRTRRSVPRDSPPQLPNFVVIYAEYCFRHRWTGRYEAHLWDKNCWNESQNKKGRQGAYDDEAAAAHAYDLAALKYWGQDTILNFPLSTYQKELKEMEDQSREEYIGSLRRKSSGFSRGVSKYRGVARHHHNGRWEARIGRVFGNKYLYLGTYATQEEAATAYDMAAIEYRGLNAVTNFDLSRYIKWLKPNQTKPENNPNPNPNIIDTTTTSLVTPNPDQELDLTFFNGNNHNQKLQESDVISETLLTQPRPVNATSALGLLLQSSKFKEMMEMTSAATDHRQSTSMISEPVRCGFPEDIQTYFECQDSSCYGNGDDLIFGELNSFGPSMFQCDQLDA

>Cotton_A_37619

MASTSSSDPGMKAEASGGENSETVIANDQLLLCRGLKKAKKERGCTAKERISKMPPCTAGKRSSIYRGVTRHRWTGRYEAHLWDKSTWNQNQNKKGKQVYLGAYDDEEAAARAYDLAALKYWGPGTLINFPVTDYTRDLEEMQNVSREDYLASLRRKSSGFSRGISKYRGLSSRRWDSSFGRVSGSEYFNSIHYGDDTTKENDYIGGFCIERKIDLTGYIKWWGTNKTRQAEAGTKSSEETKNACPEDIGSELKTSEWAVQPTEPYQMPRLGTSLEGTKCKGSSVSALSILSRSADFKSLQEKALKKQEQNSDSDENENKNTINKMDHGKAAEKSVNHDTGGDRLGAAMGMTGGLSSLQKNVYPLTPFLSAPLLTNYNTIDSLVDPVLWTSLVPALPTGPSRNPEVTKTETSSTYTFFRPEE

>Cotton_D_gene_10024797

MKRSSTCSSSSNSSIASPSSPSSPSSPSLSSSSSPSSSSSESPQNVVSVISEKPKGKRVRKNQNQKCISSNANTSRRSSIYRGVTRHRWTGRFEAHLWDKTSWNSIQNKKGRQVYLGAYDSEEDAARTYDLAALKYWGPETTLNFPVERYGKEIEEMNKVSKEEYLASLRRRSSGFSRGISKYRGVARHHHNGRWEARIGRVFGNKYLYLGTYNTEEEAAAAYDMAALEYRGVNAVTNFDISHYVERLMEKGILYLDPTPEQSPSSVEAGPIEVEQQPQQGYEAADEHQHFQNMQMQLPLCNDNATTMVGTETTDGNELAWSFCMDSGLTSFFSPEFPTVFDDMGFEDNVDSLFDLGNNKNAVGRKCLSDEASCVEVGDSSTTSVSCDGFSFWG

>Cotton_D_gene_10029828

MKRSPSCSSSSNSCFALPSPSSSSLSPSPSSSSSSSSCENPHDQSEKPKAKRARKHQNTDNNACLNNANNNGGRRSSIYRGVTRHRWTGRFEAHLWDKSSWNNIQNKKGRQGAYDSEEAAARTYDLAALKYWGAETILNFPKERYEKEMEEMKKVTKEEYLASLRRRSSGFSRGVSKYRGVARHHHNGRWEARIGRVFGNKYLYLGTYNTQEEAAAAYDMAALEYRGANAVTNFDISHYIERLKQKGILLVDRTEEQIPNPDEARRVESEENGPQPLQEQQERQEKQEQELNQEEAEKSQHFQYMQMQLPLCIDSPMTTMAGIEPTDSNELAWSFCMDSGLTSFLVPDIPLDGTAELPNLFDHDTGFEDNFDLIFDVGPPNKEEANRKCVMDDDVIGVSVSMNMEDDNRKERLSSPSSDSPCSSSTTSVSCNYSV

>Cotton_D_gene_10016054

MTKLSQVNQKNSAQSGSVNNNISTSNDVTKVKKRTRRSFPRDSPPQRSSIYRGVTRQHRWTGRYEAHLWDKNCWNESQNKKGRQVYLGAYDDEESAAHAYDLAALKYWGQDTILNFPVSTYQKELKEMENQSREEYIGSLRRKSSGFSRGVSKYRGVARHHHNGRWEARIGRVFGNKYLYLGTYATQEEAATAYDMAAIEYRGLNAVTNFDLSRYIKWLKPNQTNPTTPNSNIDATTASTLASPSHHQQHNPSFFTTTDDDNRQPQGTCVGIPSETLLTQPRPGNATSALGLLLQSSKFKEMMEMTSAVVVDQCQLTPPPSTTSEPTRYSFPENVETYLECQDSSSLADEDRDIIFGELNSFMAPMFSCDIDA

>Cotton_D_gene_10008870

MEIVTAKSEFRPGRTRLCTAEDNAIDTKCIKRRRRDHSNGALGLSNQQQQHQQLQGDQPTATTVKRSSRFRGVSRHRWTGRFEAHLWDKGSWNPTQRKKGKQGAYDEEESAARAYDLAAIKYWGTSTFTNFPVSDYGTEIEIMRSVTKEEYLASLRRRSSGFSRGVSRYRGVARHHHNGRWEARIGRVFGNKYLYLGTYSTQEEAAHAYDIAAIEYRGINAVTNFDLSTYIRWLKPGANDALISEQIKTASATRPMMTSNIFPTEQTNGLTLFNSNPLTEEAIDIRKKGVVSPCPKSSPALSLLLRSSMFNKLVEQNLNANYDQTEEKVAVDKNGGGEMLCNEVDGGVLPFMCSNNRGLESKESKVPLYNKTGQSMWNGALNLLTNA

>Cotton_D_gene_10002133

MEMMMVNKAQCLGSHRRRLCSVVDDEVQAVRCVKRRRRTPDSVAVGFDGNQGLVQVQPQQQIEQRPIAATTVKRSSRFRGVSRHRWTGRYEAHLWDKLSWNVTQKKKGKQVYLGAYDDEEAAARAYDLAALKYWGTSTFTNFSISDYEKEIEIMQTVTKEEYLASLRRRSSGFSRGVSKYRGVARHHHNGRWEARIGRVFGNKYLYLGTYSTQEEAARAYDIAAIEYRGINAVTNFDLSTYFRWLKPGTMVEPESKPSFHPSLPTDYLKSPEKQEVFKTKTPSSSSSSPTALDLLFRSSIFRELVKKNSNVSSEDGSSVTDGDDESKNQQGGNGVDADDEFSRLFYDGIGDFPFMCSSTKSSIELKHI

>Cotton_D_gene_10001570

MDMEMRMVNDERYLGLGRRRKCCDDSENVQVEAIRCAKRRRRDPAGSVAFDNRDGHQQRNAAATATTVKRSSRFRGVSRHRWTGRYEAHLWDKLSWNITQKKKGKQVYLGAYDEEEAAARAYDLAALKYWGTSTFTNFPISDYGKEIEIMQTLTKEEYLASLRRKSSGFSRGVSKYRGVARHHNNGRWEARIGRVFGNKYLYLGTYSTQEEAARAYDIAAIEYRGINAVTNFDLSTYIRWLKPNESLPMAVEPEPVTLPSQASTPREESKPSVNHSSTADYLNSFPKQVVESKIHVMNSNKCSSTTALGLLLRSSIFRDLVEKNLANVCEDESGSTDEDEEKNKHLAGNDDEFCGLFYDGIGTEFPFFRSSMKDTMELQERGSSFI

>Cotton_D_gene_10021087

MAKLSQQDHKNTTQNNTSTASNGVTKVKRTRRSVPRDSPPQRSSIYRGVTRQAIFSFFIHRWTGRYEAHLWDKNCWNESQNKKGRQGAYDDEEAAAHAYDLAALKYWGQDTILNFPLSTYQKELKEMEDQSREEYIGSLRRKSSGFSRGVSKYRGVARHHHNGRWEARIGRVFGNKYLYLGTYATQEEAATAYDMAAIEYRGLNAVTNFDLSRYIKWLKPNQTKPENNPNPNPNIIDTTTTSLVTPNPDQELDLTFFNGNNHNQQLQESDVISETLLTQPRPVNATSALGLLLQSSKFKEMMEMTSAATDHRQSTPMVSEPVRCGFPEDIQTYFECQDSSCYGNGDDLIFGELNSFGPSMFQCDQLDA

>Cotton_D_gene_10007101

MAKISHQNQKNGSGNEKTAAQPTTKLKRTRKTVPRHSPPQRSSTYRGVTRHRWTGRFEAHLWDKNCWNESQKKKGRQGAYADEEAAAHAYDLAALKYWGQDTVLNFPLATYEKELKEMESQSKEEYIGSLRRKSSGFARGVSKYRGVARHHHNGRWEARIGRVFGNKYLYLGTYATQEEAAMAYDMAAIEYRGLNAVTNFDLSRYIGWLHPNDQSDSNNSSNPQQNFNGDTNSTPSPNHDTKLEISIQSQTYCTSDTRLDDSNSNGSSSSASSALGHLLKSSKIKEMLDRTSEAACPSTPPEPNVPRRSFPDYIQTYFDCQDSSSYTEDDDIIFGDLDSLAIPMFHCELDG

>Cotton_D_gene_10014404

MEMIMVKDESYQRRRRMSSVYGDVQAVKCVKRRRRDRSDVNQGLQQNDQSSNAPAAAITVKRSSRFRGVSKHRWTGRYEAHLWDKLSWNVTQKKKGKQGAYDDEEAAARAYDLAALKYWGTSTSTNFPISDYEKEIEVMQTVTKEEFLASLRRKSSGFSRGVSKYRGVARHHHNGRWEARIGRVFGNKYLYLGTYNTQEEAARAYDIAAIEYRGINAVTNFDLSTYVGWLRPGMTNNYRIAANETPATVEPESVQSTSCYSPIEESKPSIHYPFATDYFNSPQKQQHVVETKLPVSYKSSSPTALSLLLRSSVFRELVEKNANNVSEDESSNSDADNEQKNQQSGRSDHSDEFGRLFYDEIGSGFSLFFSPTKDSIQLQENELPFVI

>Cotton_D_gene_10014405

MEMINDDNQGMQQNYQSSNVATTVKRSSRFRGVSRHRWTGKYEAHLWDKLSWNATQKKKGKQGAFDDEEVAARAYDLAALKYWGTSTSTNFPISYYEQEIEIMQTVTKEEFLASLRRKSSGFSRGVSKYRGVAKHYYHNGRWEARIGREFGNKYLNLGSYNTQEEAARAYDIAAIKCRGINAVTKFDLSTYAGWLRPELTNNTETESVQSTSCYSLIEQSKPSIHYPFSVGHFNSLKQEHAETKLPVINVKSYKSSSPTALSLLLRSSVFQELVEKNANNRK

>Cotton_D_gene_10038477

MASTSSSDPGMKAEASGGENSETVIANDQLLLCRGLKKAKKERGCTAKERISKMPPCTAGKRSSIYRGVTRHRWTGRYEAHLWDKSTWNQNQNKKGKQGAYDDEEAAARAYDLAALKYWGPGTLINFPVTDYTRDLEEMQNVSREDYLASLRRKSSGFSRGISKYRGLSSRRWDSSFGRVSGSEYFNSIHYGDDTTKENDYIGGFCIERKIDLTGYIKWWGTNKTRQAEAGTKSSEETKNACPEDIGSELKTSEWAVQPTEPYQMPRLGTSLEGTKCKGSSVSALSILSRSADFKSLQEKALKKQEQNSDNDENENKNTINKMDYGKAAEKSVNNDTGGDRLGAAMGMTGGLSSLQKNVFPLTPFLSAPLLTNYNTIDSLVDPVLWTSLVPALPTGPSRNPEVTKTETSSTYTFFRPEE
