# Supplementary material for: Genome-Scale Analysis of the WRI-Like Family in Gossypium and Functional Characterization of GhWRI1a Controlling Triacylglycerol Content
Source: Front Plant Sci. 2018 Oct 16;9:1516. doi: 10.3389/fpls.2018.01516 (PMC6198791; doi:10.3389/fpls.2018.01516)
Supplement: FILE S2 — Coding sequence of GhWRI1a. [file Data_Sheet_2.docx]

**Additional file S2:** Coding sequence of *GhWRIa*.

1 ATGAAGAGGT CACCGAGTTG TTCTTCTTCT TCTAATTCAT GCTTTGCATT GCCATCACCA

61 TCATCATCAT CATCATCACC GTCACCGTCT TCGTCATCAT CATCATCTTC ATGTGAGAAC

121 CCTCATGATC TATCAGAGAA ACCCAAGGCT AAAAGGGGTA GAAAGCATCA AAACACTGAT

181 AATAATGCTT GTTTGAACAA TGCTAACAAC AATAGCGGTA GAAGGAGCTC TATTTACAGA

241 GGAGTCACCA GGCATAGATG GACTGGGAGA TTTGAGGCTC ACCTTTGGGA CAAGAGTTCT

301 TGGAATAATA TTCAGAACAA GAAAGGAAGA CAAGTTTATT TAGGGGCTTA TGATAGTGAG

361 GAGGCAGCGG CTCGAACCTA TGATCTTGCG GCTCTCAAAT ATTGGGGGGC GGAAACGATA

421 CTGAACTTCC CGAAAGAAAG ATATGAAAAG GAGATGGAAG AAATGAAGAA AGTGACAAAG

481 GAAGAGTACT TGGCGACTCT ACGACGTCGC AGCAGTGGGT TTTCTAGAGG AGTTTCTAAG

541 TATCGTGGGG TAGCTAGGCA TCACCACAAT GGGAGGTGGG AAGCCCGAAT TGGTCGAGTT

601 TTTGGGAACA AATATCTCTA TTTAGGGACC TATAATACAC AAGAGGAAGC AGCAGCAGCA

661 TATGATATGG CAGCATTGGA GTATAGAGGG GCCAATGCCG TGACCAATTT CGATATTAGC

721 CATTACATTG AACGTTTGAA GCAGAAAGGA ATTTTGTTAG TAGATCGAAC GGAAGAACAA

781 ATTCCCAACC CCGATGAAGC TCGACGAGTA GAATCCAAAG AAAATGGACC ACAGCCGCTG

841 CAGGAGCAGC AAGAACAGCA GGAAAAACAG GAACAAGAAT TGAACCAAGA AGAGGCCGAA

901 AAATCTCAAC ATTTTCAATA CATGCAAATG CAGCTTCCTC TATGCATTGA TAGTCCGATG

961 ACAACAATGG CCGGTATTGA GCCTACTGAT AGTAATGAAC TAGCATGGAG TTTCTGCATG

1021 GATTCCGGGT TGACATCGTT TTTGGTCCCG GACATCCCTC TCGATGGAAC CGCTGAATTG

1081 CCAAACTTGT TTGATCATGA TGCGGGATTT GAGGATAACT TCGACTTGAT ATTCGACGTA

1141 GGGCCGCCTA ACAAAGAAGA GGCTAATCGG AAATGCATGA TGGATGAAGA TGTGATTGGA

1201 GTCGGTGTTT CCATGAACGT GGAAGACGAT AATAGGAAGG AGAGATTGTC ATCACTGTCT

1261 TCAGACTCTC CGTGTTCATC GACAACCTCG GTTTCTTGTA ACTACTCTGT TTAA
